# Real-world, digital-first delivery of GLP-1 receptor agonist therapy in women

**DOI:** 10.1038/s41591-026-04687-x

**Published:** 2026-10-16

**Authors:** John Deanfield, Jamie Lai Chun Wong, Michail Katsoulis, Emma Deanfield, Raj Thakkar, Riyaz Patel, Rachael Joy, Matthew Noble, Donna H. Ryan

**Affiliations:** 1 Institute of Cardiovascular Science, University College London, London, UK; 2 Unit for Lifelong Health and Ageing, Institute of Cardiovascular Science, University College London, London, UK; 3 Independent researcher, London, UK; 4 Primary Care Cardiology Lead, Oxford and Thames Valley Health Innovation Network, High Wycombe, UK; 5 Institute of Clinical Trials and Methodology, University College London, London, UK; 6 SheMed, London, UK; 7 eMed Healthcare UK, London, UK; 8 Pennington Biomedical Research Center, Louisiana State University, Baton Rouge, LA USA

**Keywords:** Health care, Risk factors

## Abstract

Glucagon-like peptide-1 receptor agonists (GLP-1RAs) have demonstrated substantial weight reduction and cardiometabolic benefit in randomized trials; however, prospective implementation evidence from digital care models and female sex community populations remains limited. Here we report outcomes from an observational study of 8,127 women initiating GLP-1RA therapy (99.8% tirzepatide) through a structured digital program incorporating mandatory biochemical monitoring, weekly video-supported follow-up and clinician-guided dose titration in the UK. Mean weight reduction was 16.1 kg (95% confidence interval 15.9 to 16.2; *P* < 0.001), representing 15.5% of baseline weight; 83.1% achieved ≥10% and 22.4% achieved ≥20% weight loss, with consistent responses across age, baseline weight and socioeconomic strata. Among participants with paired biomarker data, mean glycated hemoglobin decreased by 3.3 mmol mol^−^^1^ (95% confidence interval 3.1 to 3.4; *P* < 0.001), normalizing in 84.5% of those with baseline diabetes-range values and 96.3% with prediabetes, and lipid parameters improved significantly (reductions in total cholesterol of 0.77 mmol l^−^^1^, low-density lipoprotein cholesterol of 0.61 mmol l^−^^1^ and triglycerides of 0.47 mmol l^−^^1^). Retention was 91.4% at 6 months, with no serious treatment-related adverse events. These findings support the feasibility of delivering structured, digitally enabled obesity pharmacotherapy at scale and provide prospective real-world implementation evidence for this care model. Interpretation is limited by the observational design, absence of a comparator group, self-pay population and 6-month follow-up.

## Abstract (web summary)

At 6 months, a cohort of 8,127 women in the UK initiating GLP-1 receptor agonist therapy via a digital platform showed mean weight reduction comparable to that in randomized clinical trials.

## Main

Obesity represents a major global public health challenge and is a key driver of type 2 diabetes, cardiovascular disease, hepatic dysfunction and multiple chronic conditions that contribute to impaired quality of life and substantial healthcare and economic burden. Excess adiposity, particularly visceral and ectopic fat deposition, plays a central role in cardiometabolic disease pathogenesis, underscoring the need for effective and scalable strategies to reduce body fat and associated risk^1,2^.

Glucagon-like peptide-1 receptor agonists (GLP-1RAs) and related incretin-based therapies have transformed the therapeutic landscape for obesity management. Initially developed for diabetes treatment, these agents have demonstrated clinically meaningful weight loss, improvements in cardiometabolic risk factors and favorable clinical outcomes in large randomized trials of individuals with overweight and obesity, including those without diabetes^3–5^. The expanding population eligible for therapy presents an opportunity to reduce substantially obesity-related disease burden, but delivery at scale poses challenges for healthcare systems largely configured around in-person care models.

In many settings, access to GLP-1RAs remains restricted to individuals with advanced obesity or diabetes, contributing to increasing uptake through community and self-pay pathways^6–8^. Individuals accessing therapy through these routes may differ from trial populations with respect to comorbidity burden, treatment behaviors, dosing patterns and metabolic characteristics. Large cardiometabolic outcomes trials in obesity have also enrolled predominantly male populations despite women representing the majority of GLP-1RA users in routine and community care^6^. Digitally delivered care models offer a potential alternative framework for treatment delivery, but robust real-world evidence with this approach remains limited^9–12^. The majority of previously reported platforms operate as transactional prescribing services with limited additional features. The limitations and concerns around these approaches have been well articulated and a set of standards proposed^12^. Our program complies with these and, in addition, has several integrated and protocol-driven components that, taken together, constitute a structured clinical–digital hybrid model that more closely replicates the safety and oversight features of specialist in-person care. These include mandatory monthly structured questionnaire completion as a condition of medication refill approval, weekly live-video check-ins with trained proctors, supervised weight confirmation and medication scanning. Monthly clinician-led review enabled individualized, response-guided dose titration rather than automatic escalation. Furthermore, a proactive structured side effect mitigation package was provided alongside evolving educational content, which was available throughout the program.

We report outcomes from this large-scale digital program that delivered GLP-1RA therapy to more than 8,000 women recruited from a community-based population over a 6-month period.

The inclusive design enabled evaluation of treatment response across age, baseline weight, socioeconomic strata and ethnicity. This study is one of the largest real-world evaluations of GLP-1RA therapy in a female sex community cohort and provides evidence to complement trial findings, while informing implementation of digitally supported obesity care at scale.

## Results

### Study population

**Fig. 1:**
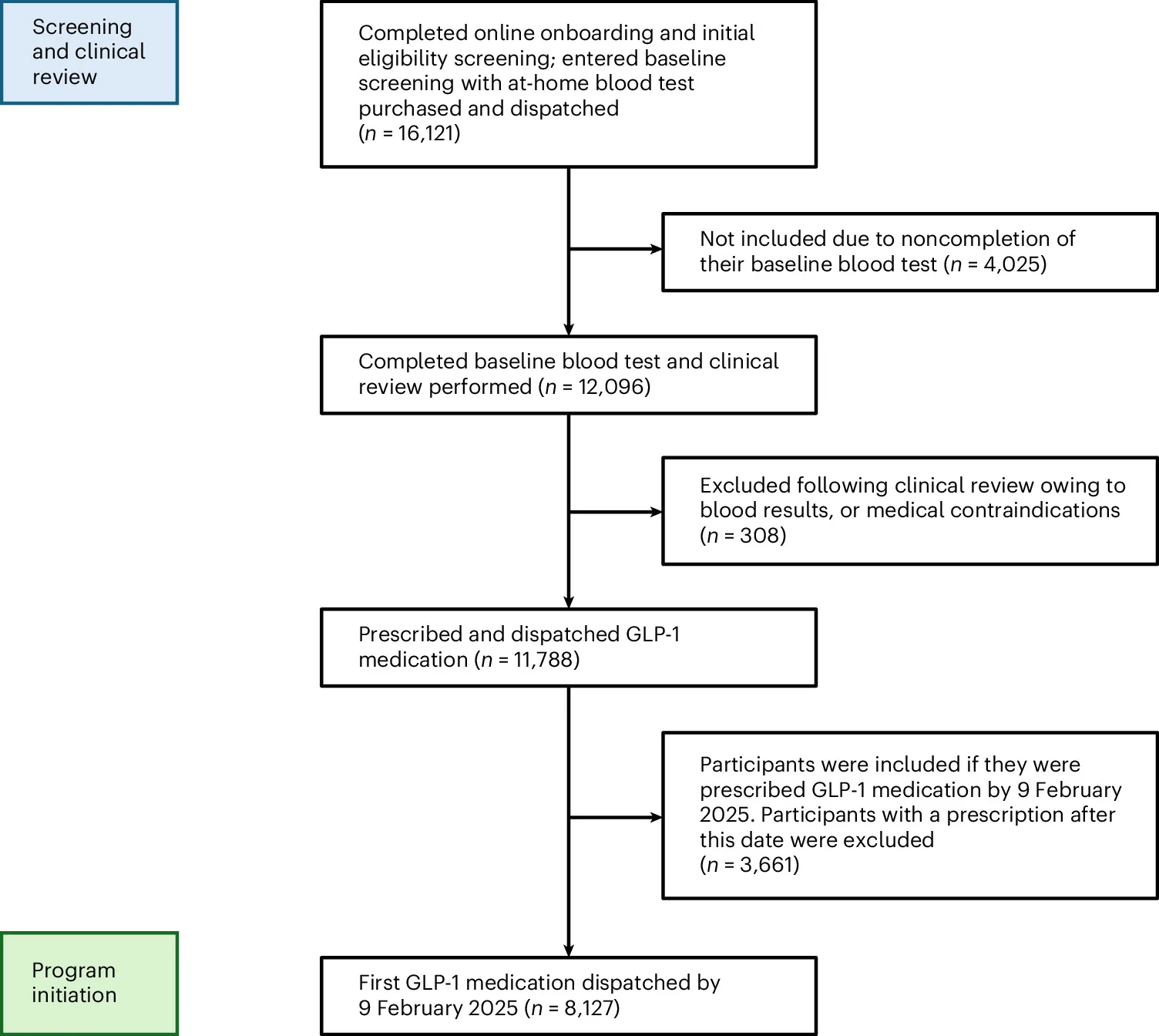
Participant flow through screening, eligibility and inclusion. A flow diagram illustrating participant recruitment, eligibility screening, baseline assessment and inclusion in the analytic cohort. Individuals were screened via a structured digital onboarding platform. Individuals were only included if they had completed baseline biochemical testing, and were excluded if clinical review identified abnormalities in blood test results or contraindications to therapy. The final analytic cohort comprised participants prescribed and dispatched GLP-1RA therapy before the administrative cutoff.

Between 27 November 2024 and 31 January 2025, adult women (female sex at birth) were recruited through digital outreach and screened via a structured online onboarding platform incorporating demographic characteristics, anthropometry, medical history, medication use and contraindications to GLP-1RA therapy (Fig. 1).

Of 16,121 individuals meeting initial screening criteria, 4,025 were not included solely owing to noncompletion of baseline blood testing, and a further 308 were excluded following clinical review, owing to abnormal laboratory findings or medical exclusion criteria. GLP-1RA therapy was prescribed and dispatched to a total of 8,127 participants before an administrative cutoff date, and they were then included in the analytic cohort (Fig. 1, Table 1 and Extended Data Table 1). This represents consecutive selection across eligibility confirmation, laboratory completion and clinical review stages within a fully remote care pathway.

**Table 1:** Baseline characteristics of the study population stratified by baseline body weight

			Baseline weight				
	Total (*N* = 8,127)	<90 kg (*N* = 2,108)		90 to <100 kg (*N* = 1,771)	100 to <110 kg (*N* = 1,495)	110 to <120 kg (*N* = 1,183)	≥120 kg (*N* = 1,570)
Sex							
Female	8,127 (100%)	2,108 (100%)		1,771 (100%)	1,495 (100%)	1,183 (100%)	1,570 (100%)
Age (years)							
18 to <30	730 (9.0%)	182 (8.6%)		158 (8.9%)	123 (8.2%)	115 (9.7%)	152 (9.7%)
30 to <40	2,096 (25.8%)	497 (23.6%)		406 (22.9%)	395 (26.4%)	330 (27.9%)	468 (29.8%)
40 to <50	2,468 (30.4%)	585 (27.8%)		564 (31.8%)	459 (30.7%)	379 (32.0%)	481 (30.6%)
50 to <60	2,107 (25.9%)	605 (28.7%)		467 (26.4%)	401 (26.8%)	259 (21.9%)	375 (23.9%)
60 to <80	726 (8.9%)	239 (11.3%)		176 (9.9%)	117 (7.8%)	100 (8.5%)	94 (6.0%)
BMI (kg m^−^^2^)							
Overweight (25.0 to <30.0)	369 (4.5%)	344 (16.3%)		24 (1.4%)	0 (0%)	0 (0%)	1 (0.1%)
Obesity I (30.0 to <35.0)	2,659 (32.7%)	1,542 (73.2%)		895 (50.5%)	195 (13.0%)	23 (1.9%)	4 (0.3%)
Obesity II (35.0 to <40.0)	2,366 (29.1%)	216 (10.2%)		775 (43.8%)	891 (59.6%)	409 (34.6%)	75 (4.8%)
Obesity III (≥40.0)	2,733 (33.6%)	6 (0.3%)		77 (4.3%)	409 (27.4%)	751 (63.5%)	1,490 (94.9%)
Total cholesterol (mmol l^−1^)							
Mean (s.d.)	5.71 (1.19)	5.80 (1.22)		5.78 (1.24)	5.79 (1.19)	5.62 (1.20)	5.51 (1.07)
Median (IQR)	5.59 (4.88–6.43)	5.67 (4.94–6.53)		5.66 (4.91–6.54)	5.67 (4.99–6.49)	5.50 (4.78–6.34)	5.43 (4.78–6.12)
Range [min, max]	1.86, 12.9	2.46, 11.4		1.86, 12.9	2.63, 10.7	2.29, 11.2	2.88, 11.8
Missing	594 (7.3%)	166 (7.9%)		128 (7.2%)	102 (6.8%)	94 (7.9%)	104 (6.6%)
HDL Cholesterol (mmol l^−1^)							
Mean (s.d.)	1.39 (0.365)	1.49 (0.403)		1.42 (0.363)	1.37 (0.345)	1.32 (0.317)	1.29 (0.325)
Median (IQR)	1.33 (1.13–1.59)	1.42 (1.20–1.71)		1.37 (1.17–1.63)	1.32 (1.12–1.56)	1.28 (1.10–1.51)	1.24 (1.07–1.45)
Range [min, max]	0.300, 3.38	0.640, 3.38		0.590, 3.36	0.600, 3.26	0.300, 2.80	0.540, 3.19
Missing	608 (7.5%)	169 (8.0%)		131 (7.4%)	101 (6.8%)	95 (8.0%)	112 (7.1%)
LDL cholesterol (mmol l^−1^)							
Mean (s.d.)	3.77 (1.08)	3.73 (1.09)		3.78 (1.15)	3.85 (1.07)	3.78 (1.11)	3.71 (0.982)
Median (IQR)	3.67 (3.02–4.42)	3.62 (2.97–4.40)		3.66 (3.01–4.49)	3.77 (3.07–4.54)	3.65 (3.05–4.44)	3.65 (3.02–4.29)
Range [min, max]	0.460, 10.2	0.770, 10.2		0.730, 9.17	1.00, 8.15	0.460, 10.1	0.800, 9.32
Missing	623 (7.7%)	169 (8.0%)		134 (7.6%)	109 (7.3%)	96 (8.1%)	115 (7.3%)
Triglycerides (mmol l^−1^)							
Mean (s.d.)	1.64 (0.847)	1.55 (0.855)		1.66 (0.894)	1.67 (0.879)	1.67 (0.815)	1.68 (0.764)
Median (IQR)	1.45 (1.07–1.97)	1.32 (0.99–1.86)		1.45 (1.07–1.97)	1.48 (1.09–1.98)	1.48 (1.12–1.98)	1.54 (1.12–2.04)
Range [min, max]	0.260, 10.4	0.370, 7.71		0.380, 7.69	0.340, 10.4	0.260, 6.78	0.530, 10.2
Missing	636 (7.8%)	172 (8.2%)		144 (8.1%)	115 (7.7%)	95 (8.0%)	110 (7.0%)
HbA1c (mmol mol^−1^)							
Mean (s.d.)	36.2 (6.13)	35.1 (5.74)		35.6 (5.32)	36.2 (5.85)	36.8 (6.28)	37.9 (7.12)
Median (IQR)	35.7 (32.9–39.0)	34.8 (32.0–37.9)		35.5 (32.6–38.4)	36.0 (33.1–38.9)	36.5 (33.6–39.6)	37.4 (34.2–40.6)
Range [min, max]	15.0, 111	16.4, 85.9		15.7, 78.4	17.1, 100	15.0, 82.6	16.1, 111
Missing	319 (3.9%)	77 (3.7%)		77 (4.3%)	62 (4.1%)	48 (4.1%)	55 (3.5%)
HbA1c category							
Diabetes (≥48 mmol mol^−1^)	206 (2.5%)	43 (2.0%)		30 (1.7%)	31 (2.1%)	35 (3.0%)	67 (4.3%)
Prediabetes (42 to <48 mmol mol^−1^)	681 (8.4%)	107 (5.1%)		120 (6.8%)	125 (8.4%)	114 (9.6%)	215 (13.7%)
Normal (<42 mmol mol^−1^)	6,921 (85.2%)	1,881 (89.2%)		1,544 (87.2%)	1,277 (85.4%)	986 (83.3%)	1,233 (78.5%)
Missing	319 (3.9%)	77 (3.7%)		77 (4.3%)	62 (4.1%)	48 (4.1%)	55 (3.5%)
Ethnicity							
Nonwhite	942 (11.6%)	348 (16.5%)		188 (10.6%)	166 (11.1%)	105 (8.9%)	135 (8.6%)
White	7,154 (88.0%)	1,750 (83.0%)		1,575 (88.9%)	1,323 (88.5%)	1,076 (91.0%)	1,430 (91.1%)
Missing	31 (0.4%)	10 (0.5%)		8 (0.5%)	6 (0.4%)	2 (0.2%)	5 (0.3%)
IMD (quintile)							
1 (most deprived)	1,354 (16.7%)	318 (15.1%)		281 (15.9%)	235 (15.7%)	214 (18.1%)	306 (19.5%)
2	1,695 (20.9%)	440 (20.9%)		350 (19.8%)	307 (20.5%)	240 (20.3%)	358 (22.8%)
3	1,753 (21.6%)	462 (21.9%)		375 (21.2%)	337 (22.5%)	261 (22.1%)	318 (20.3%)
4	1,792 (22.1%)	463 (22.0%)		416 (23.5%)	320 (21.4%)	257 (21.7%)	336 (21.4%)
5 (least deprived)	1,531 (18.8%)	424 (20.1%)		348 (19.7%)	296 (19.8%)	211 (17.8%)	252 (16.1%)
Missing	2 (0.0%)	1 (0.0%)		1 (0.1%)	0 (0%)	0 (0%)	0 (0%)

Baseline demographic, anthropometric and biochemical characteristics of participants initiating GLP-1RA therapy, presented overall and stratified by baseline body weight category. Continuous variables are summarized as mean (s.d.) and median (IQR), with ranges provided where relevant. Categorical variables are presented as counts and percentages within each column. Missing values are reported for each variable. BMI and HbA1c categories were defined according to standard clinical thresholds. Socioeconomic status was determined using nation-specific IMDs for England, Scotland, Wales and Northern Ireland, derived from participant postcodes. Biochemical measurements were obtained from capillary blood samples processed at an accredited laboratory.

### Baseline characteristics

The program was designed exclusively for adult women (female sex at birth), and sex at birth was determined through structured self-reporting during onboarding, confirmed via supervised identity verification. Table 1 and Extended Data Table 1 outline baseline participant characteristics according to weight and age groups, respectively. Mean baseline weight was 104.2 kg (s.d. of 19.8) and mean body mass index (BMI) 38.2 kg m^−^^2^ (s.d. of 6.7), with 95.5% of participants meeting criteria for class I–III obesity. Weight distribution was broad, with 19.3% of participants weighing ≥120 kg and 25.9% <90 kg. The cohort spanned a wide adult age range (18–79 years), with 82.1% aged 30 to <60 years. Mean baseline total cholesterol was 5.7 mmol l^−^^1^ (s.d. of 1.2), low-density lipoprotein (LDL) cholesterol 3.8 mmol l^−^^1^ (s.d. of 1.1) and glycated hemoglobin (HbA1c) 36.2 mmol mol^−^^1^ (s.d. of 6.1), values consistent with cardiometabolic profiles reported in community populations with obesity without established cardiovascular disease. Age-stratified analyses demonstrated modest increases in metabolic risk with advancing age, with 21.3% of participants aged ≥60 years meeting criteria for type 2 diabetes or prediabetes compared to 4.1% of those aged 18 to <30 years. Participants were predominantly white (88.0%), although representation across other ethnic groups enabled subgroup analyses of treatment response. Socioeconomic distribution was heterogeneous, with 37.6% residing within the two most deprived Index of Multiple Deprivation (IMD) quintiles. Of the 8,127 participants initiating therapy, 7,297 (89.8%) provided weight measurements at 6 months and 6,466 (79.6%) completed repeat biochemical assessment.

Baseline sociodemographic and anthropometric characteristics were broadly similar between the analytic cohort and the wider group of online registrants with valid baseline data, with no major differences in age, baseline BMI or postcode-derived IMD (Extended Data Table 2). Among participants initiating GLP-1RA therapy, baseline sociodemographic and clinical characteristics were also broadly comparable between those with and without complete paired follow-up weight or biochemical measurements, with no systematic differences between participants included in and excluded from the complete-case analytical samples (Supplementary Tables 1 and 2).

### Medication dosing and titration

**Fig. 2:**
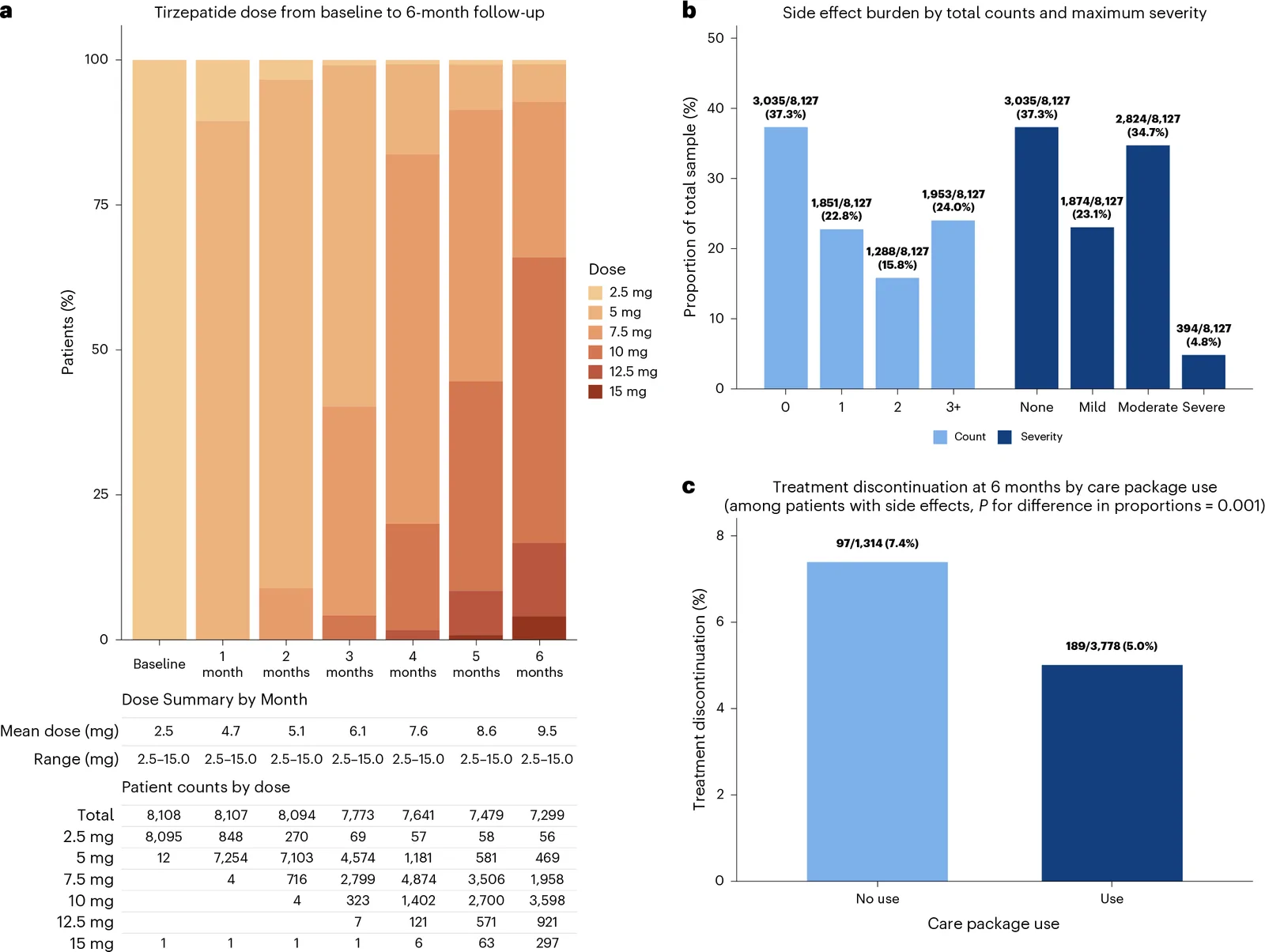
Dose exposure, side effects and treatment retention. **a**, Distribution of weekly tirzepatide dose exposure from treatment initiation to 6 months, with each monthly time point reflecting the most recent prescribed dose recorded before or during that month of follow-up. Mean dose and patient counts contributing to each time point are shown below the plot. **b**, Participant-reported side effect burden during follow-up, summarized as total count of recorded side effects per participant and maximum reported severity (none, mild, moderate or severe). Side effects were captured at medication refill intervals using structured reporting instruments. **c**, Treatment discontinuation at 6 months among participants reporting any side effects, stratified according to use of a structured symptom-mitigation package provided at treatment initiation. Semaglutide dosing trajectories are not shown owing to limited exposure (<2% of the cohort). Treatment discontinuation was defined as cessation of pharmacotherapy during the 6-month observation period.

**Extended Data Fig. 1:**
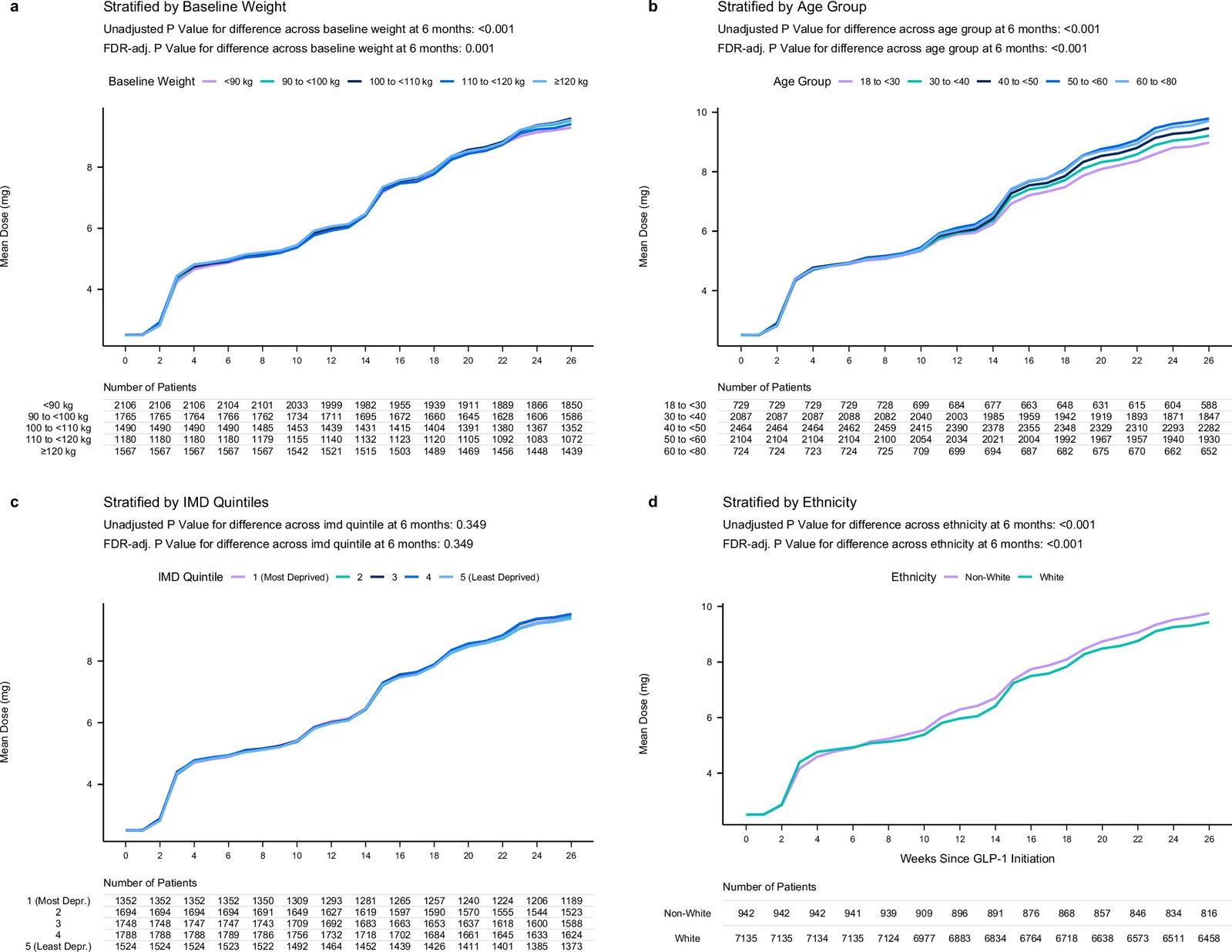
Dose trajectories across demographic strata. **a**–**d**, Mean tirzepatide dose trajectories from baseline to six months according to baseline weight category (**a**), age group (**b**), Index of Multiple Deprivation (IMD) quintile (**c**) and self-reported ethnicity (**d**). Dose at each timepoint reflects the most recent prescription recorded before or during that month of follow-up. Semaglutide trajectories are not shown owing to limited exposure within the cohort (<2% of the cohort). Benjamini-Hochberg false discovery rate (FDR) adjusted P values are presented alongside unadjusted P values to account for multiple testing, with FDR adjustment applied across the four subgroup tests shown in panels **a–d**.

Treatment initiation occurred predominantly with tirzepatide 2.5 mg weekly (99.6%), with 13 initiating at higher doses (5–15 mg). A total of 19 participants initiated with semaglutide, 17 at 0.25 mg and 2 at higher doses (0.5 or 2.4 mg). Subsequent dose escalation was guided by licensing recommendations but individualized according to weight trajectory and tolerability^13^ (Fig. 2). Clinicians were advised to defer escalation in participants demonstrating adequate weight loss (≥2 kg month^−^^1^) or reporting of clinically meaningful side effects. Mean tirzepatide exposure increased to 9.5 mg weekly at 6 months. Stratified analyses demonstrated largely parallel dose trajectories across baseline weight, age and socioeconomic strata (Extended Data Fig. 1).

### Weight outcomes

**Fig. 3:**
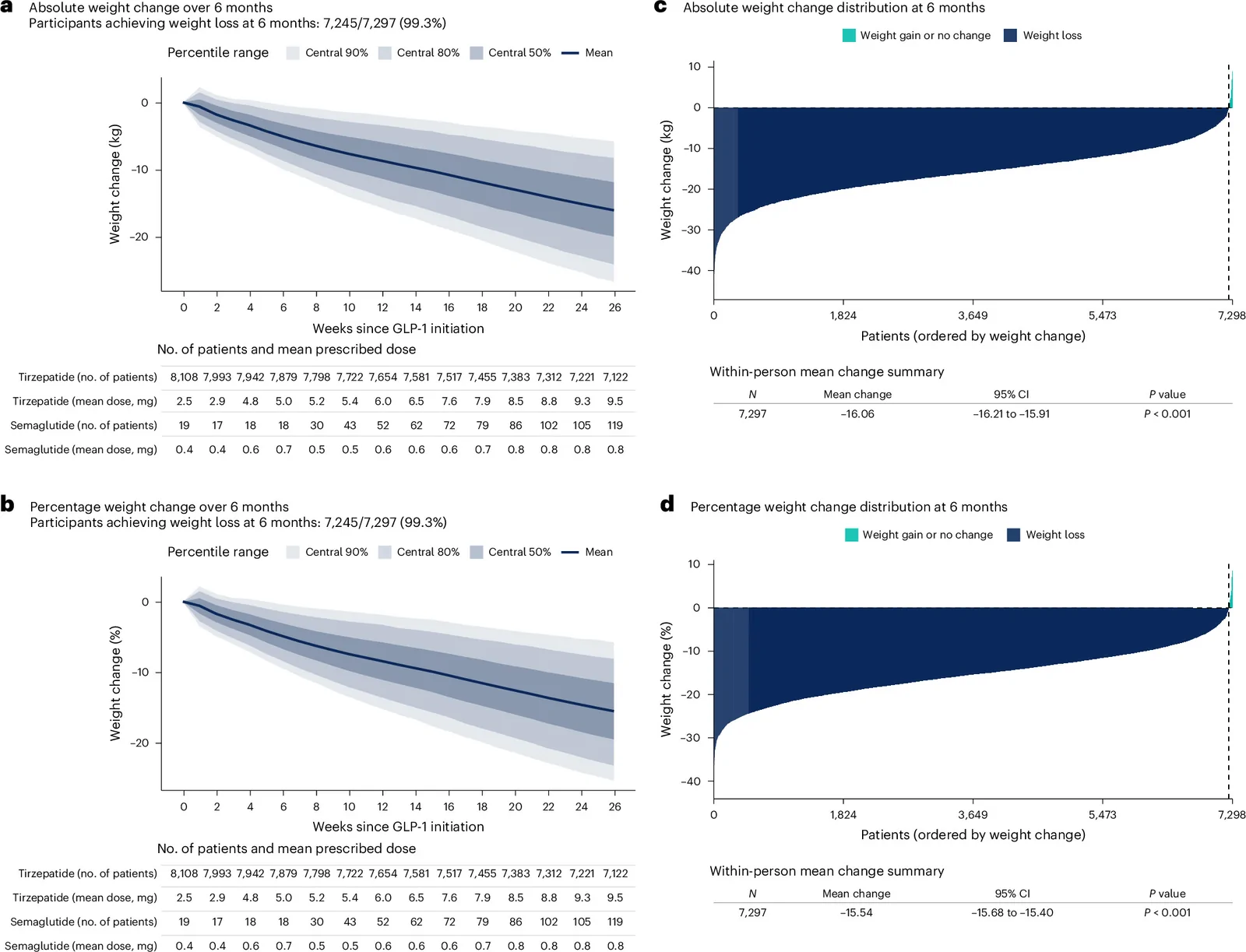
Weight trajectories during 6 months of GLP-1RA therapy. **a**,**b**, Mean absolute body weight change in kg (**a**) and percentage change (**b**) from baseline over 6 months among participants with available longitudinal weight measurements (*n* = 7,297). Values represent mean weight change at each follow-up time point following treatment initiation. Percentage weight change was calculated relative to baseline body weight for each participant. Corresponding mean medication exposure displayed within the figure was derived from each participant’s most recent prescription recorded before or during the respective follow-up time point. **c**,**d**, Absolute (**c**) and percentage (**d**) body weight change for individual participants from baseline to 6-month follow-up. Each participant’s body weight change is plotted as a single bar. *P* values were derived using paired two-sided *t*-tests comparing baseline and 6-month measurements. No adjustment for multiple comparisons was applied as these were the primary outcomes.

Among participants with available 6-month weight measurements (*n* = 7,297), 99.3% experienced weight reduction. Mean weight decreased from 104.6 kg (s.d. of 19.8) at baseline to 88.6 kg (s.d. of 19.0) at 6 months, corresponding to a mean absolute reduction of 16.1 kg (95% confidence interval (CI) 15.9 to 16.2; *P* < 0.001) or 15.5% of baseline (95% CI 15.4 to 15.7) (Fig. 3). Clinically meaningful weight loss thresholds were broadly achieved at 6 months, with 96.1% attaining ≥5% weight loss, 83.1% ≥10%, 53.5% ≥15% and 22.4% ≥20%. Within the complete-case sample, mean BMI decreased from 38.4 kg m^−^^2^ (s.d. of 6.7) to 32.5 kg m^−^^2^ (s.d. of 6.5) during the study period. At 6 months, 63.6% of participants with baseline class III obesity had transitioned to class II or lower, and 92.1% of participants with baseline class II were class I or below the obesity threshold (BMI <30 kg m^−^^2^). Temporal analyses indicated that 58.0% of total 6-month weight reduction occurred within the first 3 months.

### Weight loss heterogeneity across baseline characteristics

**Fig. 4:**
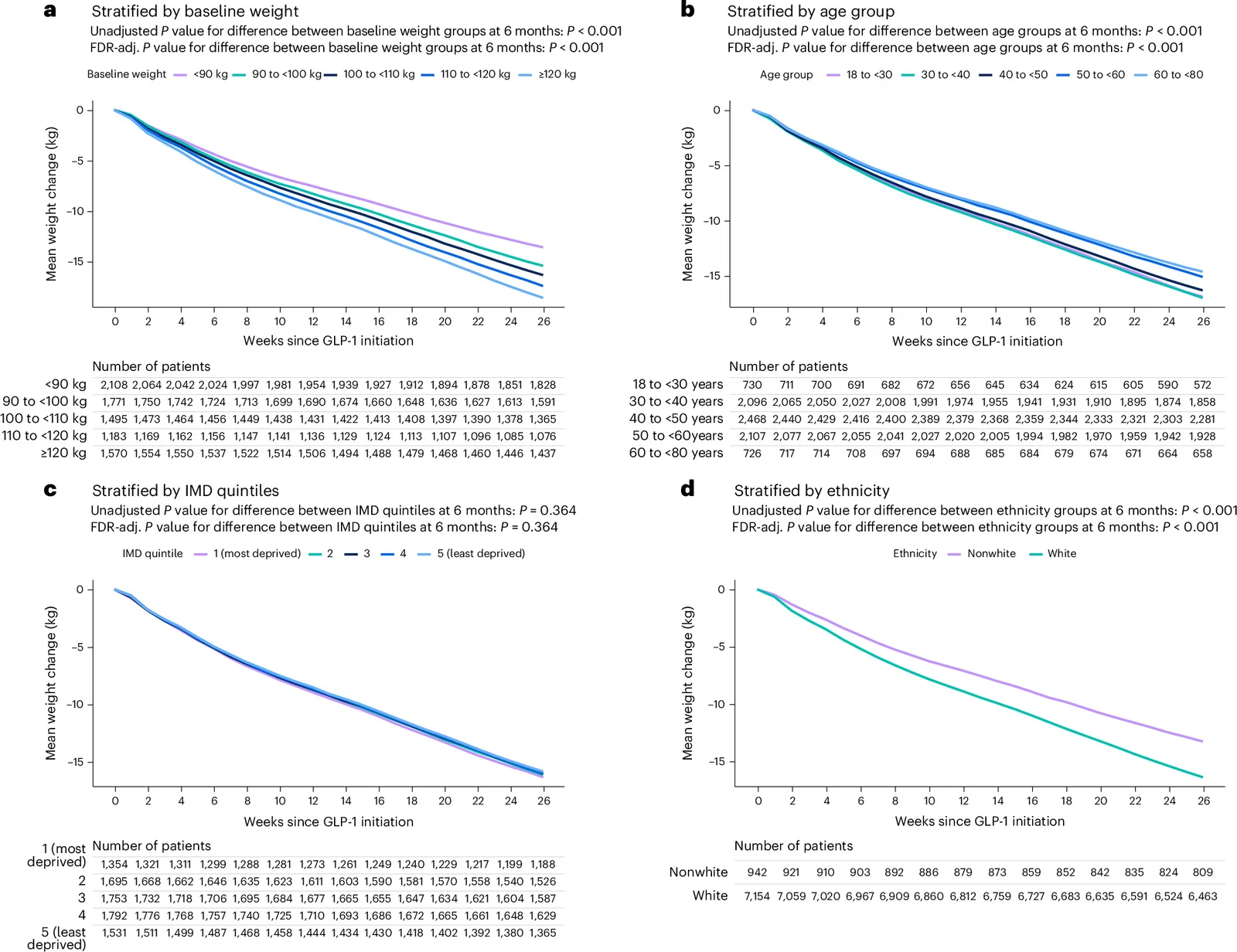
Weight-change trajectories stratified by baseline characteristics. **a**–**d**, Mean absolute weight change from baseline to 6 months following GLP-1RA initiation stratified by baseline characteristics. The graphs show trajectories according to baseline body weight category (**a**), age group (**b**), IMD quintile (**c**) and self-reported ethnicity (**d**). Values represent mean within-participant weight change at each follow-up time point. Numbers of patients at each time point for each subgroup are shown below the graphs. Between-group differences in weight change at 6 months were assessed using linear regression models, with partial *F*-tests comparing models with and without the subgroup variable. Benjamini–Hochberg FDR-adjusted (FDR-adj.) *P* values are presented alongside unadjusted *P* values to account for multiple testing, with FDR adjustment applied across the four subgroup tests shown in **a**–**d**. For **a**, baseline weight categories were defined as <90 kg, 90 to <100 kg, 100 to <110 kg, 110 to <120 kg, and ≥120 kg. For **b**, age groups were defined as 18 to <30, 30 to <40, 40 to <50, 50 to <60, and 60 to <80 years. For **c**, socioeconomic status was determined using IMD quintiles derived from participant postcode, with quintile 1 representing most deprived and quintile 5 least deprived. Analyses for each graph were restricted to participants with nonmissing data for the relevant subgroup variable.

**Extended Data Fig. 2:**
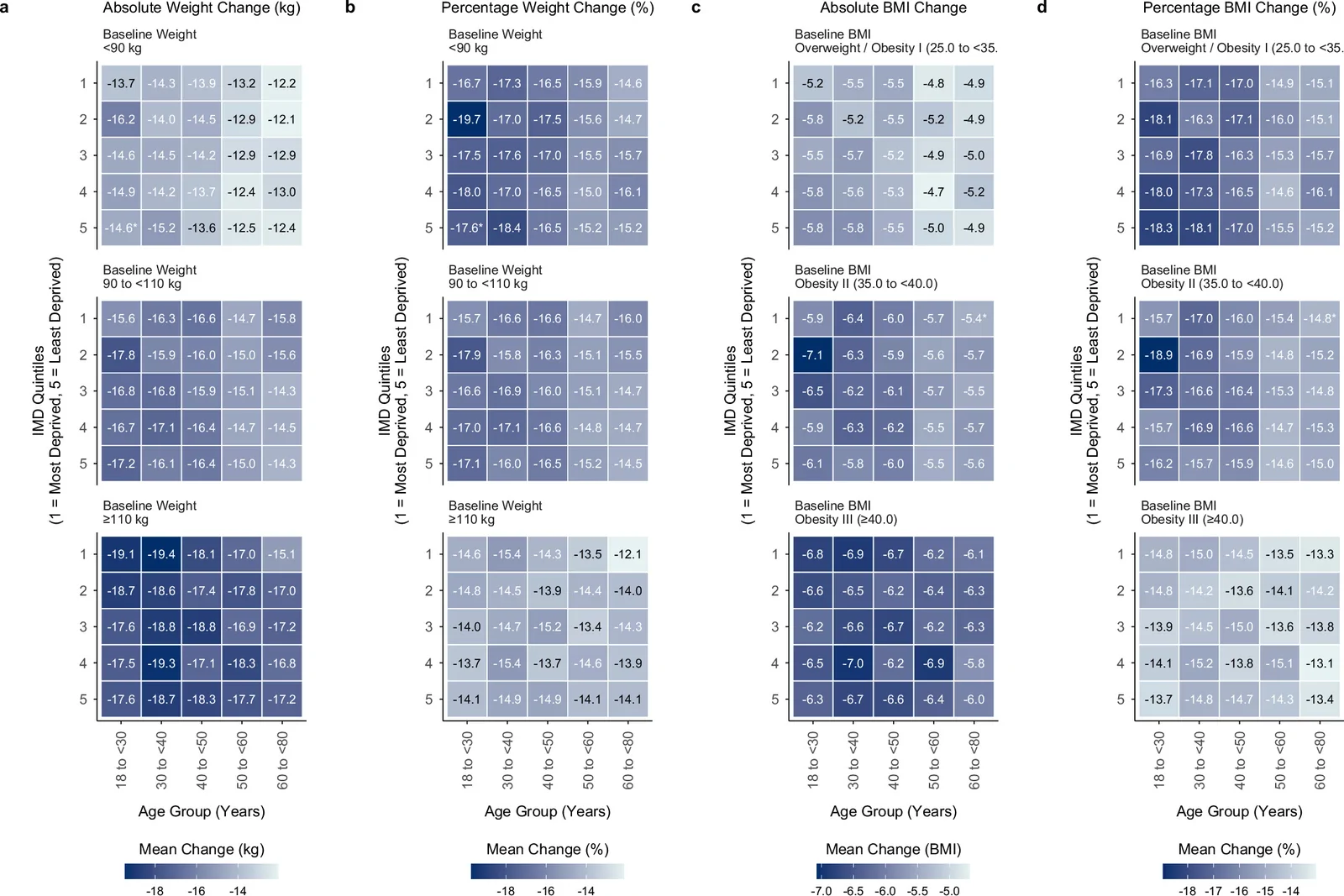
Absolute and percentage weight and BMI change across demographic strata. **a**–**d**, Heat maps displaying absolute weight change (**a**), percentage weight change (**b**), absolute BMI change (**c**) and percentage BMI change (**d**) at six months stratified by age group, Index of Multiple Deprivation (IMD) quintile and either baseline weight category (**a,b**) or baseline BMI category (**c,d**). Cells with fewer than 20 observations are suppressed and denoted by an asterisk (*). Categories were collapsed where necessary to minimise sparse cell counts.

Weight loss was observed across all baseline weight strata, with greater absolute reductions among participants with higher starting weight, but comparable percentage reductions across categories (Fig. 4). Percentage weight loss was modestly lower in older age groups (16.1% for 18 to <30 years to 14.8% for 60 to <80 years), and was modestly greater in white participants (15.8%) than in nonwhite participants (13.5%), although clinically meaningful reductions were observed in all ethnic groups (Fig. 4d). In analyses of absolute weight change, reductions did not differ significantly across IMD quintiles (*P* = 0.364; Fig. 4c). Integrated subgroup analyses, visualized as a heat map, demonstrated consistently negative weight-change estimates across combinations of baseline weight, age and deprivation strata, without evidence of subgroup nonresponse (Extended Data Fig. 2).

### Glycemic outcomes

**Fig. 5:**
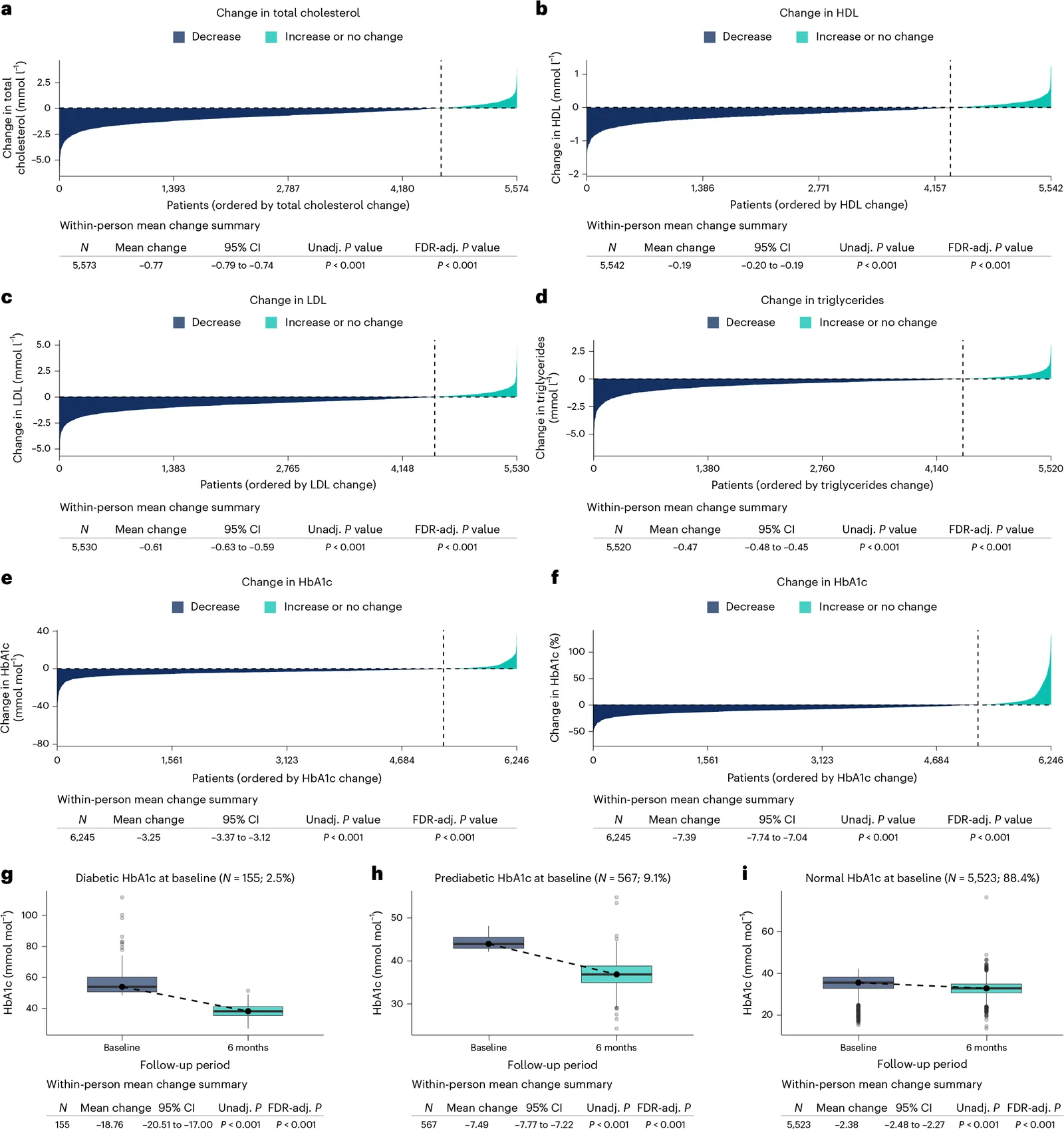
Changes in glycemic and lipid parameters during follow-up. **a**–**f**, Within-participant change in cardiometabolic biomarkers between baseline and 6 months among participants with paired measurements, with waterfall plots showing individual changes in total cholesterol (**a**), HDL cholesterol (**b**), LDL cholesterol (**c**), triglycerides (**d**) and HbA1c (expressed in mmol mol^−^^1^ (**e**) and relative percentage change from baseline (**f**)), with participants ordered according to magnitude of change. Negative values indicate reductions from baseline. **g**–**i**, Paired HbA1c distributions at baseline and 6 months stratified by baseline glycemic categories: diabetic (**g**), prediabetic (**h**) and normoglycemic (**i**). Within each box plot, the center line indicates the median, and the lower and upper bounds of the box indicate the 25th and 75th percentiles, respectively. Whiskers extend to the most extreme values within 1.5× the IQR. Values beyond the whiskers are shown as individual points, with the minimum and maximum represented by the lowest and highest points, respectively. Mean within-participant changes, 95% CIs and *P* values were derived using paired two-sided *t*-tests comparing baseline and 6-month measurements. Benjamini–Hochberg FDR-adjusted (FDR-adj.) *P* values are presented alongside unadjusted (unadj.) *P* values to account for multiple testing, with FDR adjustment applied separately within two groups of secondary outcomes: the six main blood-test outcomes are shown in **a**–**f** and the three baseline HbA1c category-specific analyses are shown in **g**–**i**. HbA1c categories were defined as normoglycemia (<42 mmol mol^−^^1^), prediabetes (42 to <48 mmol mol^−^^1^) and diabetes (≥48 mmol mol^−^^1^).

**Extended Data Fig. 3:**
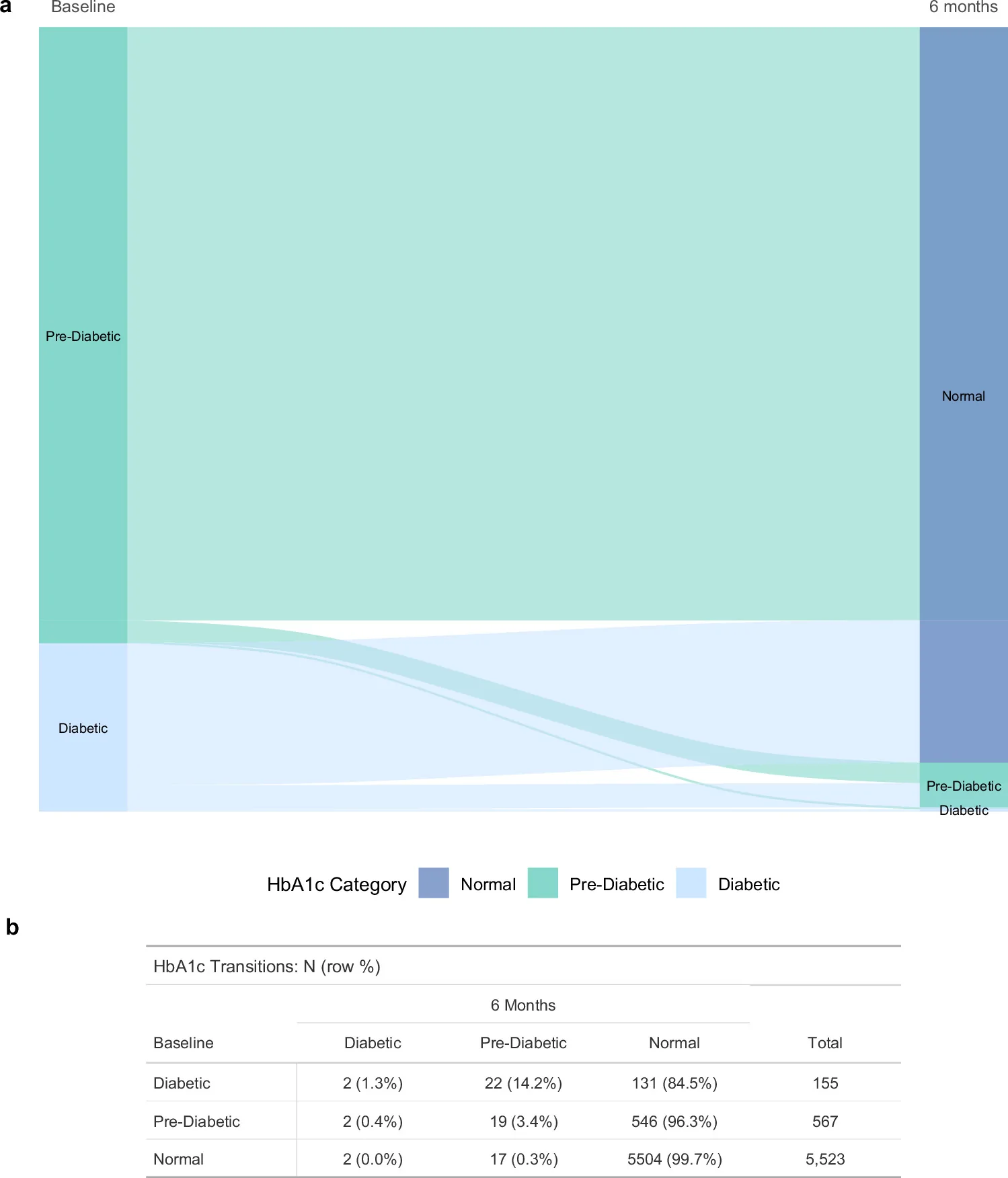
Glycaemic state transitions. **a**, Sankey diagram illustrating transitions between baseline and six-month HbA1c categories (normoglycaemia, prediabetes and diabetes). Participants with normoglycaemic HbA1c values at baseline were omitted from the visualisation to improve clarity of transition patterns. **b**, Data on transitions between baseline and six-month HbA1c categories in a tabular format. Transition counts are derived from complete-case paired measurements.

**Extended Data Fig. 4:**
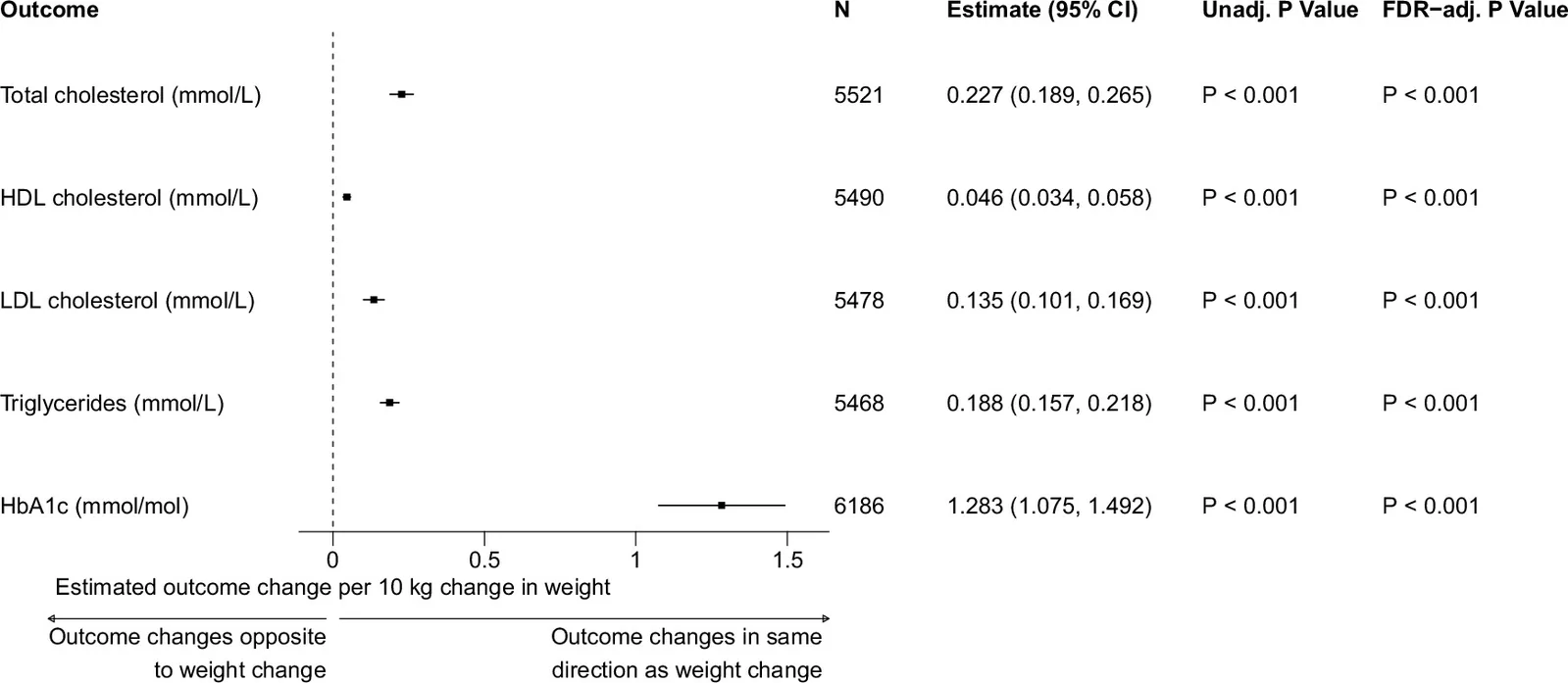
Associations between weight change and cardiometabolic biomarkers. Forest plot illustrating multivariable associations between six-month weight change and changes in HbA1c and lipid parameters among participants with paired measurements. Estimates represent mean biomarker change per 10 kg weight reduction derived from linear regression models adjusted for age, ethnicity, Index of Multiple Deprivation (IMD) quintile and baseline weight category. Benjamini-Hochberg false discovery rate (FDR) adjusted P values are presented alongside unadjusted P values to account for multiple testing across the biomarker association tests presented.

Among participants with paired HbA1c measurements (*n* = 6,245), mean HbA1c decreased from 36.3 to 33.0 mmol mol^−^^1^, equivalent to a decrease from approximately 5.5% to 5.2% on the National Glycohemoglobin Standardization Program (NGSP) scale (mean change −3.3 mmol mol^−^^1^; *P* < 0.001) (Fig. 5). At baseline, 155 participants (2.5%) exhibited HbA1c values within the diabetic range (≥48 mmol mol^−1^) and 567 (9.1%) within the prediabetic range (42 to <48 mmol mol^−1^). Following 6 months of therapy, HbA1c normalized in 84.5% (131/155) of participants with baseline diabetic-range values and 96.3% (546/567) of those with prediabetes, while progression from normoglycemia to dysglycemia occurred in only 0.3% of participants (19/5,523) (Extended Data Fig. 3). Adjusted linear regression modeling indicated that each 10-kg greater weight reduction was associated with a 1.3 mmol mol^−^^1^ greater decrease in HbA1c (Extended Data Fig. 4).

### Lipid outcomes

Among participants with paired lipid measurements, significant improvements were observed across lipid parameters (Fig. 5). Mean total cholesterol decreased by 0.77 mmol l^−^^1^ (95% CI 0.74 to 0.79; *P* < 0.001), LDL cholesterol by 0.61 mmol l^−^^1^ (95% CI 0.59 to 0.63; *P* < 0.001) and triglycerides by 0.47 mmol l^−^^1^ (95% CI 0.45 to 0.48; *P* < 0.001). High-density lipoprotein (HDL) cholesterol demonstrated a modest reduction of 0.19 mmol l^−^^1^(95% CI 0.19 to 0.20; *P* < 0.001). Multivariable regression analyses demonstrated graded associations between weight reduction and lipid improvement, with each 10 kg of weight loss associated with reductions in total cholesterol of 0.23 mmol l^−^^1^ (95% CI 0.19 to 0.27; *P* < 0.001) and LDL cholesterol of 0.14 mmol l^−^^1^ (95% CI 0.10 to 0.17; *P* < 0.001) (Extended Data Fig. 4).

### Concomitant medication use

Data on baseline medication history and newly started concomitant medication during study follow-up were available for 8,091 of 8,127 participants. Overall, 165 participants (2.0%) reported any diabetic agent at baseline or during follow-up, predominantly metformin, and 436 participants (5.4%) reported any cholesterol-lowering medication, predominantly statins. Newly started diabetic and cholesterol-lowering medications were infrequently reported during follow-up. Although medication data were self-reported, these findings suggest that changes in glucose or lipid-lowering therapies were unlikely to explain fully the observed HbA1c and lipid changes.

### Engagement, safety and adherence

**Extended Data Fig. 5:**
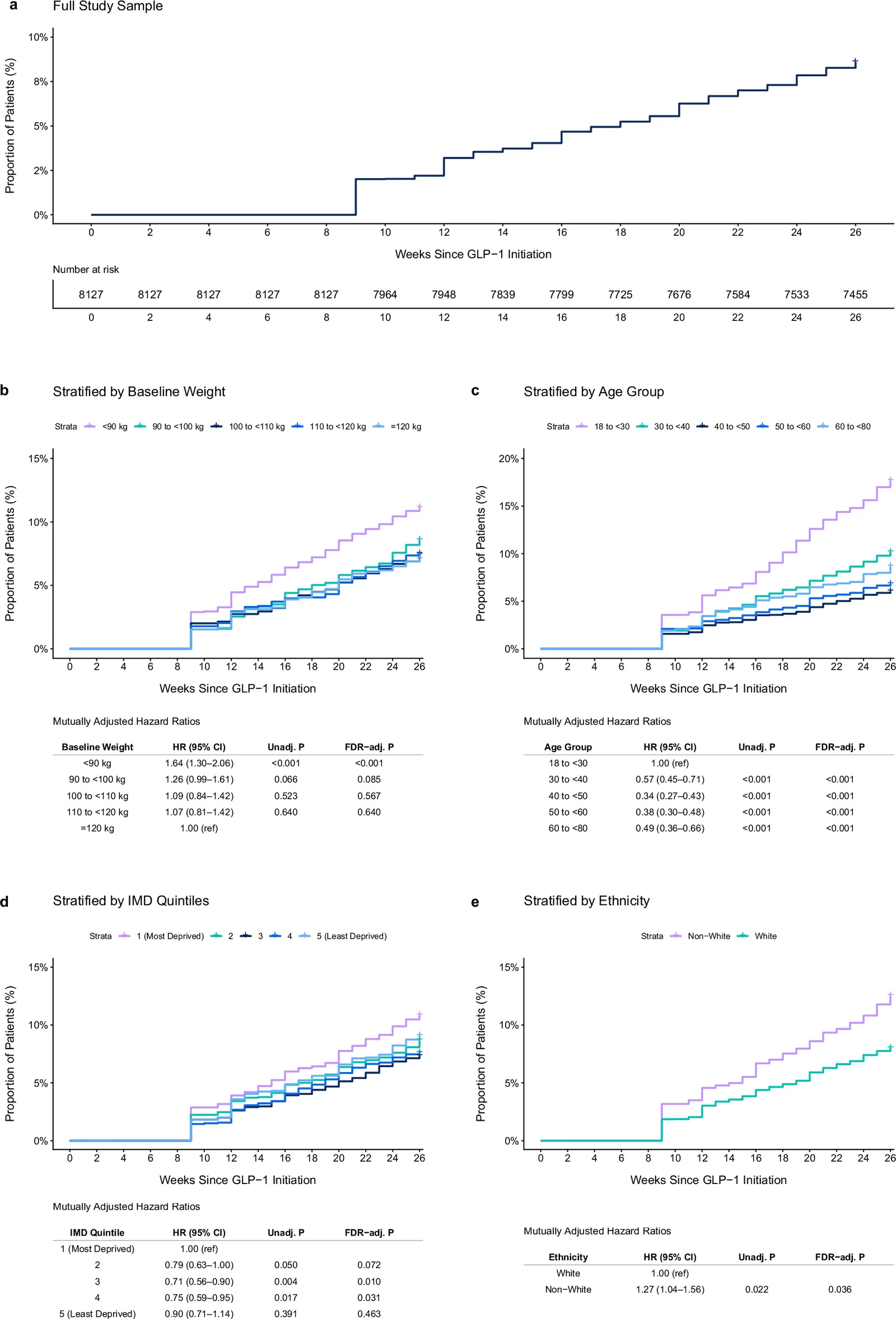
Cumulative incidence of treatment discontinuation. **a**–**e**, Kaplan–Meier curves depicting cumulative incidence of treatment discontinuation from baseline to six months overall (**a**) and stratified by baseline weight category (**b**), age group (**c**), Index of Multiple Deprivation (IMD) quintile (**d**) and self-reported ethnicity (**e**). Hazard ratios were estimated using mutually adjusted Cox proportional hazards models controlling for age, ethnicity, IMD quintile and baseline weight category. Subgroup analyses were restricted to participants with complete covariate data. Benjamini-Hochberg false discovery rate (FDR) adjusted P values are presented alongside unadjusted P values for the mutually adjusted hazard ratios to account for multiple testing.

Engagement with the digital program remained high throughout follow-up. The 6-month biochemical reassessment was completed by 6,466 of 8,127 participants (79.6%), and 7,429 (91.4%) remained on treatment at 6 months, with 698 (8.6%) discontinuing therapy (Extended Data Fig. 5). In multivariable Cox regression analyses, older age groups demonstrated lower hazards of discontinuation compared to those aged 18 to <30 years (hazard ratio (HR) range 0.34–0.57; all *P* < 0.001). Relative to participants in the most deprived IMD quintile, those in quintiles 3 and 4 exhibited lower hazards (HR range 0.71–0.75; all *P* ≤ 0.05), while no significant difference was observed for quintiles 2 and 5, suggesting a nonmonotonic association rather than a clear socioeconomic gradient. Participants with baseline weight <90 kg had higher hazards of discontinuation compared to those weighing ≥120 kg (HR 1.64, 95% CI 1.30 to 2.06; *P* < 0.001), while other weight categories did not differ significantly. Nonwhite participants had higher hazards of discontinuation compared to white participants (HR 1.27, 95% CI 1.04 to 1.56; *P* < 0.05) (Extended Data Fig. 5).

**Extended Data Fig. 6:**
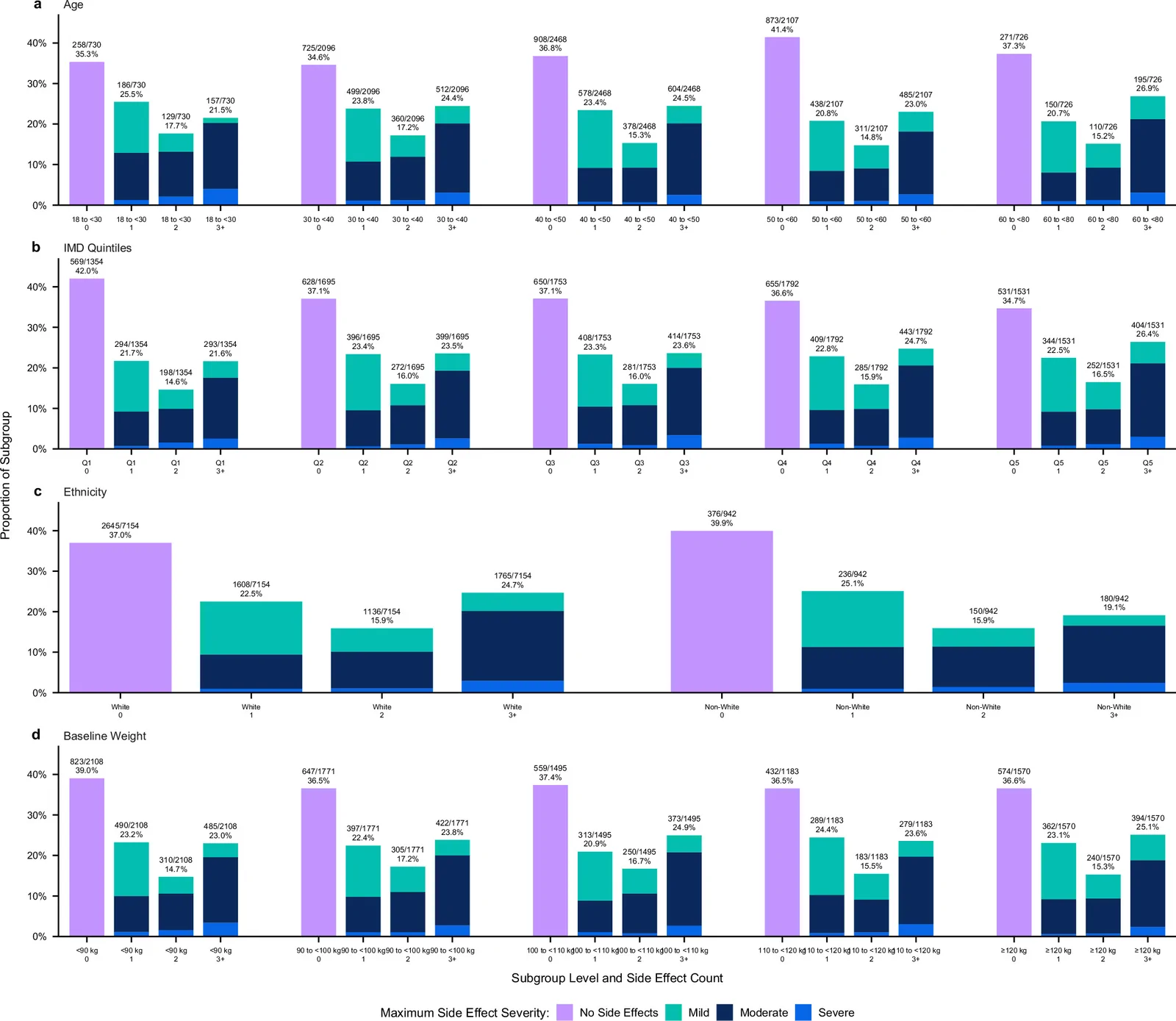
Side effect burden across demographic subgroups. **a**–**d**, Distribution of participant-reported side effect counts and maximum severity across age groups (**a**), Index of Multiple Deprivation (IMD) quintiles (**b**), self-reported ethnicity (**c**), and baseline weight categories (**d**). Side effects were reported at medication refill intervals using structured questionnaires. Severity categories were defined as none, mild, moderate or severe.

Overall, 5,092 of 8,127 participants (62.7%) reported at least one side effect during follow-up. Among these 5,092 participants, the maximum reported side effect severity was mild in 36.8%, moderate in 55.4% and severe in 7.7% (Fig. 2b), and little variation was present across baseline weight categories or age groups (Extended Data Fig. 6). Among participants who discontinued treatment, 286 of 698 (41.0%) reported at least one side effect, and 39 (5.6%) reported those as severe. Use of the recommended symptom-mitigation strategy was associated with lower discontinuation compared to nonuse (189 of 3,778 (5.0%) versus 97 of 1,314 (7.4%); *P* = 0.001) (Fig. 2c). During follow-up, 11 of 8,127 participants reported pregnancy, all of whom discontinued GLP-1RA therapy immediately. No serious treatment-related adverse events were identified.

### Determinants of treatment response

**Extended Data Fig. 7:**
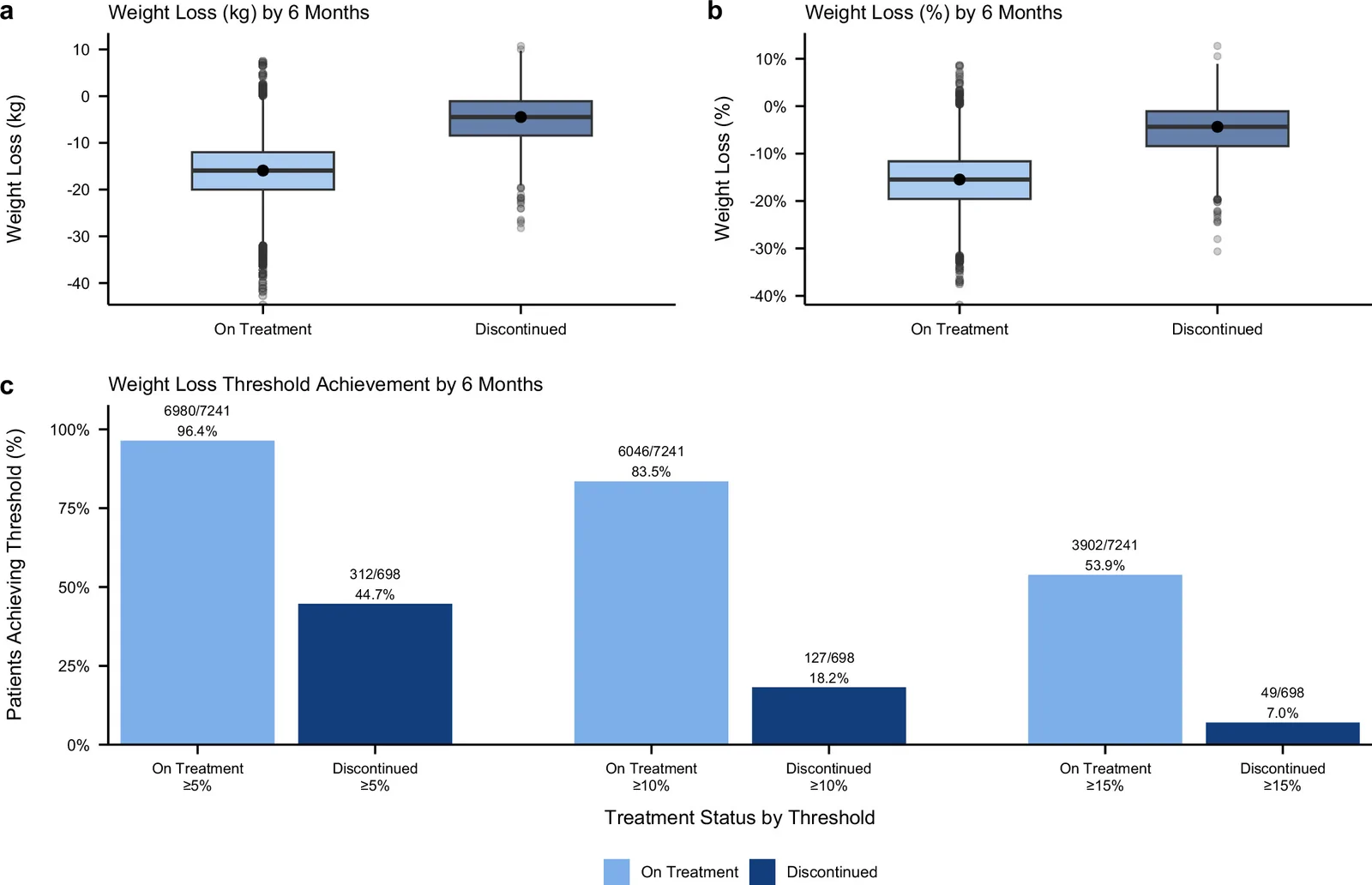
Weight loss according to treatment discontinuation status. **a,b**, Distribution of absolute (**a**) and percentage (**b**) weight change at final available follow-up among participants who discontinued treatment and those who completed six months of follow-up. Within each box plot, the centre line indicates the median, and the lower and upper bounds of the box indicate the 25th and 75th percentiles respectively. Whiskers extend to the most extreme values within 1.5 times the interquartile range. Values beyond the whiskers are shown as individual points, with the minimum and maximum represented by the lowest and highest points respectively. **c**, Weight loss threshold attainment according to clinically meaningful weight loss thresholds (≥5%, ≥10%, ≥15%) by six months is summarised by discontinuation status.

Exploratory subgroup analyses demonstrated consistent weight reduction across combinations of baseline weight, age and socioeconomic status, without evidence of subgroup nonresponse (Extended Data Fig. 2). There were no patterns associated with both reduced effectiveness and increased discontinuation. Participants discontinuing treatment before 6 months achieved smaller overall weight reduction than those completing follow-up. Among participants with paired data, completers were substantially more likely to achieve ≥5% weight loss than those who discontinued before 6 months (6,980 of 7,241 (96.4%) versus 312 of 698 (44.7%)). At final available follow-up, mean weight loss was 16.1 kg (15.6%) among completers compared to 5.4 kg (5.4%) among discontinuers. However, nearly half of discontinuers (44.7%) had achieved ≥5% weight loss at their last recorded measurement, indicating that many had derived clinically meaningful benefit before cessation (Extended Data Fig. 7). Analyses of early weight trajectories showed no evidence that discontinuation was preceded by greater initial weight loss. Comparative analyses between tirzepatide and semaglutide exposure did not demonstrate significant differences in weight loss, although interpretation is limited by the small number of semaglutide-treated participants.

### Sensitivity analyses

Sensitivity analyses supported the robustness of the overall findings. Inverse-probability-weighted analyses of primary and secondary weight and biochemical outcomes produced estimates similar to those from the complete-case analyses. Models predicting outcome completeness had *c*-statistics ranging from 0.600 to 0.684, and there was no evidence of extreme values or instability in the stabilized weights. For secondary and exploratory analyses involving multiple related comparisons, Benjamini–Hochberg false discovery rate (FDR) adjustment did not alter the overall interpretation of the findings (Extended Data Table 3).

## Discussion

In this large real-world cohort of women receiving GLP-1RA therapy within a digitally delivered care model, treatment was associated with substantial weight loss, broad cardiometabolic improvement and sustained program engagement over 6 months. The study population differed in several important respects from participants enrolled in pivotal randomized trials, including exclusive female sex representation, community-based recruitment and delivery outside conventional healthcare settings. These findings extend the evidence for incretin-based therapies beyond trial environments and demonstrate that clinically meaningful benefit can be achieved within a scalable, remote framework of obesity care.

The magnitude of weight reduction observed in this cohort was consistent with early phase trajectories reported in tirzepatide and semaglutide trials, despite lower mean and maximal dosing exposure^3,4^. Mean percentage weight loss of 15.5% at 6 months aligns with early phase trajectories observed in STEP-1 and SURMOUNT-1, and temporal analyses demonstrated that approximately 58.0% of total weight reduction occurred within the first 3 months, consistent with known early pharmacodynamic response patterns from trials^3,4^. There was near-universal weight loss (>99%), contrasting with heterogeneity and nonresponse clusters reported in some routine-care datasets^11^.

An important observation was the achievement of weight reduction consistent with early phase trajectories reported in tirzepatide and semaglutide randomized trials but at lower dosing levels. Mean tirzepatide exposure at 6 months was 9.5 mg weekly, substantially below maximal licensed doses, indicating that effective weight reduction was frequently achieved without escalation to higher doses. Additional analyses demonstrated broadly similar dosing trajectories across age, baseline weight and socioeconomic strata, supporting the feasibility of response-guided titration within digitally delivered care at scale.

Metabolic improvements in glycemic and lipid parameters support the clinical relevance of weight reduction achieved within this program. HbA1c reductions were accompanied by marked transitions from dysglycemic to normoglycemic states, with >80% of participants with baseline diabetes-range HbA1c values and >95% with prediabetes achieving normalization at 6 months. Lipid improvements were directionally consistent across parameters and demographic strata, and regression analyses demonstrated graded associations between weight reduction and biomarker change, indicating coordinated cardiometabolic adaptation.

The modest reduction in HDL cholesterol observed at 6 months is consistent with the transient decreases during the active weight-loss phase in dietary and pharmacologically mediated weight loss. Longer-duration incretin trials have demonstrated subsequent HDL increases following weight stabilization, showing that short-term HDL trajectories may differ from longer-term patterns. These findings are consistent with trial evidence that demonstrates broad metabolic and cardiovascular benefit across populations with obesity and cardiometabolic disease^5,14–17^.

Our present study provides insight into implementation and engagement within a digitally delivered pharmacotherapy program. Retention exceeded 90% at 6 months, which compares favorably with discontinuation rates in routine clinical practice and administrative database analyses^10,11^. There was modest variation in discontinuation risk across demographic strata without clear clinical determinants of cessation. The association between deprivation and treatment discontinuation was nonmonotonic, with lower hazards in the middle IMD quintiles but similar hazards in the most and least deprived groups, and therefore did not indicate a simple socioeconomic gradient. Furthermore, discontinuation was not associated with early weight-loss magnitude or side effect burden, suggesting that factors beyond treatment response and tolerability may contribute to treatment persistence in community settings. Although reasons for discontinuation could not be definitively established, these findings highlight the importance of considering behavioral, contextual and structural determinants of long-term pharmacotherapy engagement.

Side effects were predominantly gastrointestinal and largely mild or moderate, consistent with established safety profiles of GLP-1RAs^9^. Participants who used the structured symptom-mitigation package demonstrated lower discontinuation rates and favorable symptom distributions, suggesting potential value of proactive side effect management strategies within remote treatment pathways.

Our study focused exclusively on adult women (female sex at birth). Large cardiometabolic outcomes trials of incretin-based therapies have enrolled predominantly male populations despite the high prevalence of obesity among female individuals and substantial sex differences in adiposity distribution, metabolic risk and treatment utilization^6^. Our study therefore addresses an important evidence gap and demonstrates consistent effectiveness across age, baseline weight and socioeconomic strata. Although the cohort was predominantly white, there was a broadly parallel response across ethnic groups, but this warrants confirmation in prespecified populations. Differences in adiposity phenotype, hormonal milieu and cardiometabolic risk trajectories in female individuals may modulate GLP-1RA pharmacological responses, yet they remain poorly evaluated in clinical trials. Our cohort spanned reproductive, perimenopausal and postmenopausal age ranges, and the consistency of weight loss across these age groups (Fig. 4b) and overall metabolic improvement in the cohort (Fig. 5) suggest that menopause-associated metabolic transitions do not substantially attenuate treatment response. In addition, a direct comparison of outcomes between male and female individuals within the same digitally delivered framework would provide further information on sex-specific treatment effects.

These real-world findings should be considered in the context of rapidly expanding global utilization of incretin-based therapies. Randomized trials have established these agents as a transformative therapeutic class, demonstrating substantial weight reduction together with favorable effects across cardiovascular, renal and metabolic outcomes^5,14–17^. Because of this, delivering at the scale required remains challenging. There is wide variation across healthcare systems in workforce capacity, prescribing infrastructure and access to populations who would benefit^1,2,7,18^. Digitally mediated care models may provide a potential mechanism to extend access, while maintaining safety and effectiveness; however, empirical evidence describing outcomes within such frameworks remains limited. Our study provides real-world support for digitally mediated care models to enable population-scale delivery of incretin-based pharmacotherapy outside traditional healthcare settings. Previous reports have used platforms that operate as transactional prescribing services with limited structured monitoring or safety oversight, and have often been small, retrospective and/or focused on populations with diabetes^13,19^. Our program is distinguished from these by the addition of several integrated and protocol-driven components that have not been previously implemented in combination. The digital infrastructure (remote eligibility assessment, app-based longitudinal monitoring, home biomarker testing and clinician-supervised response-guided titration) is technology-agnostic and transferable, although prospective evaluation in diverse regulatory and health systems will be required to establish broad international generalizability. We have demonstrated robust effectiveness across socioeconomic strata, suggesting that digitally delivered pharmacotherapy need not exacerbate health inequalities when appropriately designed. Furthermore, the combination of remote biomarker monitoring, structured behavioral support and clinician oversight illustrates the potential for hybrid clinical–digital models to replicate key components of specialist care without requiring frequent in-person interaction. The link between weight loss and metabolic improvement reinforces the importance of early treatment access as a strategy for cardiometabolic disease prevention.

Our study has limitations inherent to observational real-world research. Causal attribution to pharmacotherapy cannot be definitively established, and residual confounding is possible. Self-reported data, including medical history and medications, were collected but not systematically verified against electronic health and prescription records. However, reported use of glucose- and lipid-lowering therapies was uncommon, and newly started medications in these classes were infrequently reported during follow-up. This makes it unlikely that concomitant medication changes fully accounted for the observed HbA1c and lipid outcomes, although residual misclassification or unreported medication changes cannot be excluded.

Participation in a self-pay program introduces an inherent economic access barrier, and willingness to pay for a structured health program may itself select individuals with higher health motivation, contributing to high retention rates and favorable outcomes. The subsidized pricing in our study may not be a sustainable, scalable commercial structure. The broad IMD distribution of the cohort, however, suggests that the pricing and digital-access model was not exclusionary, and the economics of GLP-1RA therapy will be transformed by both novel oral therapies and future loss of patent protection.

Although follow-up completeness was high, some outcome data were unavailable, and main analyses were conducted using complete-case approaches for each endpoint. Baseline characteristics were broadly similar between participants with and without complete paired data, and inverse-probability-weighted estimates were materially similar to the complete-case results. Models predicting outcome completeness showed modest discrimination. However, these analyses assume that outcome missingness was missing at random, conditional on the observed covariates, and cannot exclude bias from missing not at random mechanisms.

Some missingness in biochemical outcomes may have occurred through the use of home capillary blood collection rather than supervised venous sampling. This may have contributed to incomplete paired measurements owing to collection and transport constraints but greatly facilitated the collection of biochemical data. Additional measurements, such as blood pressure and waist circumference, represent important directions for future work as they are key cardiometabolic risk markers, and novel drug combinations aiming to improve the ratio of fat to muscle loss in response to GLP-1RA therapy are being developed. Our current report is limited to 6 months, which captures the early phase trajectory of GLP-1RA-mediated weight loss. Longer-term evaluation will be required to characterize durability, adherence trajectories and post-treatment weight dynamics, particularly given evidence that discontinuation of incretin-based therapy is frequently associated with weight regain and reversal of metabolic benefit^20,21^. Our study population was predominantly healthy with limited comorbidity, and the findings should not be generalized to populations who require GLP-1RA therapy owing to greater obesity-related complications. Notwithstanding these considerations, strengths include the scale of the cohort, high engagement and comprehensive cardiometabolic phenotyping without requiring clinic attendance.

In conclusion, GLP-1RA therapy delivered within a structured digital program was associated with clinically meaningful weight loss, metabolic improvement and high treatment retention in a large community-based cohort of women. These findings suggest that digitally enabled care models may provide a viable and scalable strategy to extend access to incretin-based pharmacotherapy as demand for obesity treatment continues to expand.

## Methods

### Study design and setting

This was a real-world, observational cohort study of adult women (female sex at birth) enrolled in a digital GLP-1RA weight-management program delivered in the UK. It was designed to evaluate whether a structured digital delivery model of drugs with established efficacy could achieve clinically meaningful outcomes at scale in a community population. The program provided remote eligibility assessment, clinician review, electronic prescribing and longitudinal monitoring without routine in-person visits (see the Supplementary Methods for further details). The study population comprised individuals recruited between 27 November 2024 and 31 January 2025 who met predefined eligibility criteria, initiated GLP-1RA therapy (tirzepatide or semaglutide) by 9 February 2025 and contributed follow-up data within a 6-month observation period following treatment initiation.

The program operated as a direct-access digital service in which participants self-enrolled and paid a fixed fee covering pharmacotherapy, laboratory testing and access to digital monitoring and clinical support components. No additional payments were required for study participation beyond routine program costs. The program operated independently of the present analysis, which was conducted retrospectively using routinely collected service data captured prospectively during program delivery. The program was partially subsidized by the provider during the study period.

### Eligibility criteria

Eligibility criteria included female individuals aged 18–80 years and with BMI ≥30 kg m^−^^2^, or BMI 27–30 kg m^−^^2^ with a qualifying weight-related comorbidity (including type 2 diabetes, prediabetes, hypertension, dyslipidemia, asthma/chronic obstructive pulmonary disease on regular inhaled corticosteroids, polycystic ovary syndrome, obstructive sleep apnea and heart disease) or ethnicity-specific BMI threshold consistent with National Institute for Health and Care Excellence guidance (South Asian, Chinese, other Asian, Black African, African-Caribbean or Middle Eastern)^7^. Exclusion criteria comprised GLP-1RA use within the preceding 12 months, contraindicated medical conditions or medications, and pregnancy or breastfeeding (see the Supplementary Methods for further details).

Eligible individuals underwent identity verification and supervised weight confirmation during video onboarding by a nonmedical proctor and completed an at-home baseline capillary blood test before treatment initiation. Inability to complete onboarding procedures and failure to confirm a baseline weight measurement at supervised video onboarding resulted in exclusion.

### Digital care program and clinical management

The program was delivered entirely through secure digital platforms without clinic attendance. Participants completed remote onboarding structured eligibility questionnaires, identity verification via supervised video consultation and at-home baseline capillary blood testing using a validated home collection kit (Randox). Submitted clinical information and laboratory data were reviewed by the program’s clinical team to confirm treatment suitability in accordance with UK prescribing regulations and the program’s clinical governance framework.

Eligible participants were prescribed GLP-1RA therapy by UK-registered clinicians practicing within a Care Quality Commission-registered service operated by eMed Healthcare UK Limited. Medication selection was informed by clinical judgment and participant preference. Treatment initiation and dose escalation followed licensed titration schedules, with individualized adjustments guided by weight trajectory, tolerability and clinician assessment at monthly review intervals. Clinical oversight was provided by a pooled team of 25 general practitioners, with support from clinical pharmacists and advanced nurse practitioners.

Following treatment initiation, participants interacted with the program primarily through a smartphone application enabling remote weight reporting, health questionnaire completion and the synchronous monthly clinical communication, aligned with medication refills. Weight entries were prompted weekly, and questionnaires captured weight trajectory, side effect burden and participant-reported well-being. Nonclinical program staff were available throughout to support engagement, facilitate data collection and provide technical assistance; they did not deliver clinical advice. At medication refills, participants were also asked to report pregnancy or suspected pregnancy. Participants reporting pregnancy during follow-up were instructed to discontinue GLP-1RA therapy immediately and were referred for appropriate clinical follow-up.

Participants could additionally report side effects between scheduled contacts using an in-app reporting function. A structured symptom-mitigation package targeting common gastrointestinal side effects associated with GLP-1RA therapy was provided to all participants at treatment initiation. The package included over-the-counter medication, recommendations and dietary guidance on dietary modification, delivered through the app. Reports of severe or concerning symptoms triggered clinical review within 24 h of receipt, with escalation according to prespecified program safety protocols. Participants were advised to seek emergency care for symptoms consistent with acute serious illness and were provided with guidance regarding emergency service contact. A detailed description is provided in the Supplementary Methods.

### Data collection

Demographic characteristics, including sex at birth, age, ethnicity and residential postcode, were self-reported during onboarding. Eligibility was restricted to participants reporting female sex at birth. Socioeconomic status was measured using area-level deprivation based on the IMD, a multidimensional measure of relative neighborhood deprivation in the UK. As participants resided across all four UK nations, the most recent nation-specific IMD for England, Wales, Scotland and Northern Ireland was assigned according to participants’ postcodes (see the Supplementary Methods for further details). Body weight was measured remotely using participant-owned digital scales. Participants were instructed to measure weight under standardized conditions (for example, light clothing, no shoes, same participant-owned digital scales and measurement at the same time of day where feasible) (see the Supplementary Methods for further details). Weight values were recorded to the nearest 0.1 kg. Verification procedures included supervised video confirmation, and, where required, photographic evidence and repeated measures. Height was self-reported and used to calculate BMI. Blood pressure was not collected as part of the digital program’s routine data capture during the study period and was therefore not available for analysis. Capillary blood sampling kits were provided for home collection of HbA1c and lipid measurements at baseline and 6 months. Samples were processed by Randox Clinical Laboratory Service, a UK Accreditation Service-accredited laboratory. Participants were instructed to return samples on the day of collection using prepaid priority postal packaging compliant with biological substance transport regulations. Side effects were captured through structured questionnaires administered at monthly intervals and spontaneous participant reports to the proctoring and/or clinical team. Reports of severe or concerning symptoms triggered clinical review within 24 h and escalation according to program safety protocols. Medication history, including concomitant medication use, was collected at baseline before prescribing. Refill questionnaires also captured concomitant medications initiated since the previous prescription for GLP-1RA therapy.

### Endpoints

The primary effectiveness endpoint was change in body weight from baseline to 6 months following treatment initiation, expressed as both absolute (kg) and percentage change. Secondary effectiveness endpoints included the proportion of participants achieving clinically meaningful weight-loss thresholds (≥5%, ≥10%, ≥15% and ≥20% of baseline weight) and changes in metabolic parameters over the same period. Glycemic outcomes were assessed using HbA1c measured at baseline and 6 months, with transitions between normoglycemia (<42 mmol mol^−^^1^), prediabetes (42 to <48 mmol mol^−^^1^) and diabetes (≥48 mmol mol^−^^1^) evaluated. Lipid outcomes included within-participant changes in total cholesterol, LDL cholesterol, HDL cholesterol and triglycerides. Program engagement and tolerability were assessed through treatment discontinuation during follow-up and participant-reported side effects. Treatment discontinuation was defined as absence of a recorded GLP-1RA dispensing event for >8 weeks following the most recent dispense. Safety outcomes included frequency and severity of reported side effects during the observation period.

### Statistical analysis

Program data were pseudonymized before transfer to an independent statistical analysis team. Prespecified data-quality procedures identified implausible laboratory values and physiologically inconsistent weight measurements using predefined rules described in the Supplementary Methods. Baseline characteristics were summarized using means with standard deviations (s.d.), medians with interquartile ranges (IQRs) and counts with percentages as appropriate. Within-participant changes in continuous outcomes from baseline to 6 months were analyzed using paired *t*-tests. Differences in 6-month weight change and GLP-1 dosing across baseline subgroups were evaluated using linear regression models with nested model comparison using partial *F*-tests. Treatment discontinuation according to symptom-mitigation package use was examined using Pearson’s *χ*^2^ tests, and time-to-event analyses using multivariable Cox proportional hazards regression adjusted for age, ethnicity, IMD quintile and baseline weight category. Complete-case analyses were performed for each endpoint using participants with paired baseline and 6-month follow-up measurements. As sensitivity analyses, primary and secondary paired change estimates were repeated using stabilized inverse probability weighting to assess the potential influence of missing baseline or follow-up outcome data. The discrimination of the models used to derive the inverse probability weights was assessed using the *c*-statistic (see the Supplementary Methods for further details). For secondary outcomes and exploratory analyses involving multiple related hypothesis tests, Benjamini–Hochberg FDR adjustment was applied within predefined groups of related comparisons presented within the same figure, with FDR-adjusted *P* values presented alongside unadjusted *P* values. All tests were two-sided, with significance defined as *P* < 0.05. Analyses were conducted using R version 4.5.2.

### Ethics

Participants provided electronic consent during program onboarding for use of their data for service evaluation and research purposes. Data were pseudonymized before transfer for analysis. In accordance with UK Health Research Authority guidance, the study constituted secondary analysis of routinely collected service data and did not require research ethics committee review. The study was conducted in accordance with the Declaration of Helsinki and applicable data-protection legislation.

### Reporting summary

Further information on research design is available in the Nature Portfolio Reporting Summary linked to this article.

## Online content

Any methods, additional references, Nature Portfolio reporting summaries, source data, extended data, supplementary information, acknowledgements, peer review information; details of author contributions and competing interests; and statements of data and code availability are available at https://doi.org/10.1038/s41591-026-04687-x.

## Supplementary information

Supplementary Information Supplementary Methods and Tables 1 and 2.

Reporting Summary

Peer Review File

## Data Availability

Owing to the sensitive participant-level demographic and health information contained in the dataset, the data cannot be made openly available. Data and an accompanying data dictionary will be available for 5 years from publication to qualified researchers affiliated with recognized institutions, subject to applicable regulatory requirements and institutional approval. Requests should include the research objectives, methods, details of ethical approval and required data and be submitted to support@shemed.co.uk. Requests will be assessed within 6 months for scientific validity. Approved applicants will receive de-identified participant-level data after signing a data sharing agreement covering data security, permitted use and onward sharing.

## Extended data

**Extended Data Table 1:** Baseline age-stratified characteristics

		Age Group				
	Total (N=8127)	18 to <30 (N=730)	30 to <40 (N=2096)	40 to <50 (N=2468)	50 to <60 (N=2107)	60 to <80 (N=726)
**Sex**						
Female	8127 (100%)	730 (100%)	2096 (100%)	2468 (100%)	2107 (100%)	726 (100%)
**Weight (kg)**						
<90 kg	2108 (25.9%)	182 (24.9%)	497 (23.7%)	585 (23.7%)	605 (28.7%)	239 (32.9%)
90 to <100 kg	1771 (21.8%)	158 (21.6%)	406 (19.4%)	564 (22.9%)	467 (22.2%)	176 (24.2%)
100 to <110 kg	1495 (18.4%)	123 (16.8%)	395 (18.8%)	459 (18.6%)	401 (19.0%)	117 (16.1%)
110 to <120 kg	1183 (14.6%)	115 (15.8%)	330 (15.7%)	379 (15.4%)	259 (12.3%)	100 (13.8%)
≥120 kg	1570 (19.3%)	152 (20.8%)	468 (22.3%)	481 (19.5%)	375 (17.8%)	94 (12.9%)
**BMI (kg/m** ^2^ **)**						
Overweight (25.0 to <30.0)	369 (4.5%)	43 (5.9%)	93 (4.4%)	95 (3.8%)	103 (4.9%)	35 (4.8%)
Obesity I (30.0 to <35.0)	2659 (32.7%)	227 (31.1%)	651 (31.1%)	798 (32.3%)	714 (33.9%)	269 (37.1%)
Obesity II (35.0 to <40.0)	2366 (29.1%)	200 (27.4%)	574 (27.4%)	747 (30.3%)	636 (30.2%)	209 (28.8%)
Obesity III (≥40.0)	2733 (33.6%)	260 (35.6%)	778 (37.1%)	828 (33.5%)	654 (31.0%)	213 (29.3%)
**Total Cholesterol (mmol/L)**						
Mean (SD)	5.71 (1.19)	5.08 (0.981)	5.39 (1.06)	5.72 (1.09)	6.14 (1.23)	6.07 (1.42)
Median (IQR)	5.59 (4.88, 6.43)	4.98 (4.41, 5.68)	5.29 (4.66, 6.02)	5.62 (4.98, 6.37)	6.06 (5.28, 6.90)	5.92 (5.09, 6.99)
Range [Min, Max]	1.86, 12.9	1.86, 9.75	2.29, 11.2	2.60, 12.9	2.65, 11.7	2.99, 11.5
Missing	594 (7.3%)	37 (5.1%)	111 (5.3%)	137 (5.6%)	212 (10.1%)	97 (13.4%)
**HDL Cholesterol (mmol/L)**						
Mean (SD)	1.39 (0.365)	1.23 (0.296)	1.30 (0.329)	1.39 (0.350)	1.50 (0.382)	1.53 (0.394)
Median (IQR)	1.33 (1.13, 1.59)	1.19 (1.01, 1.38)	1.24 (1.06, 1.48)	1.33 (1.15, 1.58)	1.44 (1.22, 1.72)	1.47 (1.25, 1.74)
Range [Min, Max]	0.300, 3.38	0.600, 3.19	0.610, 3.37	0.300, 3.13	0.540, 3.38	0.690, 3.26
Missing	608 (7.5%)	35 (4.8%)	114 (5.4%)	139 (5.6%)	218 (10.3%)	102 (14.0%)
**LDL Cholesterol (mmol/L)**						
Mean (SD)	3.77 (1.08)	3.39 (0.916)	3.60 (1.02)	3.78 (1.02)	4.01 (1.14)	3.89 (1.30)
Median (IQR)	3.67 (3.02, 4.42)	3.33 (2.81, 3.98)	3.52 (2.90, 4.22)	3.69 (3.09, 4.40)	3.91 (3.21, 4.73)	3.72 (2.96, 4.73)
Range [Min, Max]	0.460, 10.2	0.800, 8.52	0.750, 10.2	0.460, 8.71	0.730, 9.32	1.29, 9.17
Missing	623 (7.7%)	38 (5.2%)	116 (5.5%)	141 (5.7%)	224 (10.6%)	104 (14.3%)
**Triglycerides (mmol/L)**						
Mean (SD)	1.64 (0.847)	1.41 (0.751)	1.53 (0.820)	1.65 (0.843)	1.79 (0.875)	1.75 (0.856)
Median (IQR)	1.45 (1.07, 1.97)	1.21 (0.910, 1.67)	1.34 (0.980, 1.83)	1.46 (1.08, 1.96)	1.59 (1.19, 2.13)	1.56 (1.16, 2.10)
Range [Min, Max]	0.260, 10.4	0.340, 5.32	0.260, 10.2	0.380, 10.4	0.460, 8.17	0.380, 7.71
Missing	636 (7.8%)	40 (5.5%)	119 (5.7%)	150 (6.1%)	225 (10.7%)	102 (14.0%)
**HbA1c (mmol/mol)**						
Mean (SD)	36.2 (6.13)	34.3 (4.82)	35.1 (5.88)	36.0 (5.99)	37.2 (6.21)	38.9 (6.78)
Median (IQR)	35.7 (32.9, 39.0)	34.4 (31.6, 37.3)	34.9 (32.0, 37.9)	35.6 (32.8, 38.8)	36.7 (33.9, 39.9)	38.3 (35.3, 41.4)
Range [Min, Max]	15.0, 111	15.0, 61.4	16.1, 111	15.7, 91.6	16.9, 100	16.7, 82.3
Missing	319 (3.9%)	27 (3.7%)	80 (3.8%)	105 (4.3%)	87 (4.1%)	20 (2.8%)
**HbA1c Category**						
Diabetes (≥48 mmol/mol)	206 (2.5%)	3 (0.4%)	33 (1.6%)	59 (2.4%)	65 (3.1%)	46 (6.3%)
Pre-Diabetes (42 to <48 mmol/mol)	681 (8.4%)	27 (3.7%)	127 (6.1%)	166 (6.7%)	252 (12.0%)	109 (15.0%)
Normal (<42 mmol/mol)	6921 (85.2%)	673 (92.2%)	1856 (88.5%)	2138 (86.6%)	1703 (80.8%)	551 (75.9%)
Missing	319 (3.9%)	27 (3.7%)	80 (3.8%)	105 (4.3%)	87 (4.1%)	20 (2.8%)
**Ethnicity**						
Non-White	942 (11.6%)	157 (21.5%)	352 (16.8%)	252 (10.2%)	144 (6.8%)	37 (5.1%)
White	7154 (88.0%)	567 (77.7%)	1738 (82.9%)	2203 (89.3%)	1959 (93.0%)	687 (94.6%)
Missing	31 (0.4%)	6 (0.8%)	6 (0.3%)	13 (0.5%)	4 (0.2%)	2 (0.3%)
**Index of Multiple Deprivation (Quintile)**						
1 (Most Deprived)	1354 (16.7%)	163 (22.3%)	417 (19.9%)	404 (16.4%)	298 (14.1%)	72 (9.9%)
2	1695 (20.9%)	187 (25.6%)	479 (22.9%)	501 (20.3%)	380 (18.0%)	148 (20.4%)
3	1753 (21.6%)	134 (18.4%)	438 (20.9%)	537 (21.8%)	446 (21.2%)	198 (27.3%)
4	1792 (22.1%)	138 (18.9%)	430 (20.5%)	550 (22.3%)	522 (24.8%)	152 (20.9%)
5 (Least Deprived)	1531 (18.8%)	108 (14.8%)	330 (15.7%)	476 (19.3%)	461 (21.9%)	156 (21.5%)
Missing	2 (0.0%)	0 (0%)	2 (0.1%)	0 (0%)	0 (0%)	0 (0%)

Baseline demographic, anthropometric and biochemical characteristics of participants initiating GLP-1 receptor agonist therapy, presented overall and stratified by baseline age groups. Continuous variables are summarised as mean (s.d.) and median (interquartile range), with ranges provided where relevant. Categorical variables are presented as counts and percentages within each column. Missing values are reported for each variable. Body mass index (BMI) and HbA1c categories were defined according to standard clinical thresholds. Socioeconomic status was determined using nation-specific Indices of Multiple Deprivation (IMD) for England, Scotland, Wales and Northern Ireland, derived from participant postcodes. Biochemical measurements were obtained from capillary blood samples processed at an accredited laboratory. HDL, high-density lipoprotein; LDL, low-density lipoprotein; HbA1c, glycated haemoglobin; IMD, Index of Multiple Deprivation.

**Extended Data Table 2:** Baseline characteristics of all online registrants stratified by inclusion in the final analytical sample

		Sample	
	Total (N=16022)	Included (N=8127)	Not Included (N=7895)
**Sex**			
Female	16022 (100%)	8127 (100%)	7895 (100%)
**Age**			
Mean (SD)	45.9 (11.3)	45.0 (10.9)	46.9 (11.6)
Median (IQR)	46.0 (37.0, 55.0)	45.0 (37.0, 53.0)	47.0 (38.0, 56.0)
Range [Min, Max]	19.0, 83.0	19.0, 80.0	19.0, 83.0
**BMI (kg/m** ^2^ **)**			
Healthy (18.5 to <25.0)	15 (0.1%)	0 (0%)	15 (0.2%)
Overweight (25.0 to <30.0)	1010 (6.3%)	355 (4.4%)	655 (8.3%)
Obesity I (30.0 to <35.0)	5195 (32.4%)	2576 (31.7%)	2619 (33.2%)
Obesity II (35.0 to <40.0)	4430 (27.6%)	2385 (29.3%)	2045 (25.9%)
Obesity III (≥40.0)	5364 (33.5%)	2811 (34.6%)	2553 (32.3%)
Missing	8 (0.1%)	0 (0%)	8 (0.1%)
**Index of Multiple Deprivation (Quintile)**			
1 (Most Deprived)	2752 (17.2%)	1349 (16.6%)	1403 (17.8%)
2	3322 (20.7%)	1689 (20.8%)	1633 (20.7%)
3	3472 (21.7%)	1757 (21.6%)	1715 (21.7%)
4	3535 (22.1%)	1800 (22.1%)	1735 (22.0%)
5 (Least Deprived)	2928 (18.3%)	1530 (18.8%)	1398 (17.7%)
Missing	13 (0.1%)	2 (0.0%)	11 (0.1%)

Baseline demographic and anthropometric characteristics are shown for all individuals who registered on the SheMed website and provided at least one valid baseline response for sex, age, BMI, or postcode-derived Index of Multiple Deprivation (IMD), which were the only baseline characteristics collected at initial online sign-up. Characteristics are presented overall and stratified according to inclusion in the final analytical sample. Individuals were included in the analytical sample if they fulfilled eligibility criteria, initiated GLP-1 receptor agonist therapy on or before 9 February 2025, the treatment initiation cutoff for study inclusion (please see Fig. 1 for further details). Continuous variables are summarised as mean (standard deviation) and median (interquartile range), with ranges provided where relevant. Categorical variables are presented as counts and percentages within each column. The distributions of variables differ slightly from the main baseline characteristics table because these data were self-reported at initial website sign-up, whereas the main baseline table uses values recorded immediately before treatment initiation. For some participants, there was a delay of several months between initial registration and treatment initiation while eligibility checks, blood tests, and clinical review were completed. A further 99 registrants had no valid baseline responses for any of the variables shown in this table and were therefore excluded from this summary, giving a total of 16,022 registrants rather than 16,121.

**Extended Data Table 3:** Inverse probability weighted sensitivity analysis of primary and secondary outcome estimates

Subgroup	Outcome	Measure	Complete-Case Estimate			IPW Estimate				IPW Diagnostics	
			Mean Change (95% CI)	P	FDR-adj. P	Mean Change (95% CI)	P	FDR-adj. P	C-statistic	Mean (Min, Max)	Percentiles (5th, 25th, 50th, 75th, 95th)
**Primary Weight Outcomes**											
Full Sample	Weight (kg)	Absolute Change	-16.06 (-16.21, -15.91)	<0.001		-16.03 (-16.18, -15.88)	<0.001		0.624	1.000 (0.898, 1.570)	0.953, 0.967, 0.984, 1.010, 1.111
Full Sample	Weight (% change)	Percent Change	-15.54 (-15.68, -15.40)	<0.001		-15.56 (-15.70, -15.42)	<0.001		0.624	1.000 (0.898, 1.570)	0.953, 0.967, 0.984, 1.010, 1.111
**Secondary Blood Test Outcomes**											
Full Sample	HbA1c (mmol/mol)	Absolute Change	-3.25 (-3.37, -3.12)	<0.001	<0.001	-3.20 (-3.33, -3.08)	<0.001	<0.001	0.676	0.961 (0.870, 1.983)	0.883, 0.906, 0.933, 0.981, 1.131
Full Sample	HbA1c (% change)	Percent Change	-7.39 (-7.74, -7.04)	<0.001	<0.001	-7.28 (-7.63, -6.92)	<0.001	<0.001	0.676	0.961 (0.870, 1.983)	0.883, 0.906, 0.933, 0.981, 1.131
Full Sample	Total Cholesterol (mmol/L)	Absolute Change	-0.77 (-0.79, -0.74)	<0.001	<0.001	-0.76 (-0.79, -0.74)	<0.001	<0.001	0.601	1.001 (0.859, 2.660)	0.876, 0.913, 0.965, 1.050, 1.240
Full Sample	HDL (mmol/L)	Absolute Change	-0.19 (-0.20, -0.19)	<0.001	<0.001	-0.19 (-0.20, -0.19)	<0.001	<0.001	0.602	1.001 (0.858, 2.611)	0.873, 0.912, 0.969, 1.054, 1.240
Full Sample	LDL (mmol/L)	Absolute Change	-0.61 (-0.63, -0.59)	<0.001	<0.001	-0.61 (-0.63, -0.59)	<0.001	<0.001	0.601	1.001 (0.855, 2.642)	0.873, 0.911, 0.968, 1.053, 1.240
Full Sample	Triglycerides (mmol/L)	Absolute Change	-0.47 (-0.48, -0.45)	<0.001	<0.001	-0.46 (-0.48, -0.44)	<0.001	<0.001	0.600	1.001 (0.859, 2.570)	0.873, 0.912, 0.967, 1.053, 1.243
**Secondary HbA1c Outcomes by Baseline Category**											
Baseline Diabetic (≥48 mmol/mol)	HbA1c (mmol/mol)	Absolute Change	-18.76 (-20.51, -17.00)	<0.001	<0.001	-18.58 (-20.38, -16.79)	<0.001	<0.001	0.665	0.997 (0.790, 1.853)	0.803, 0.883, 0.947, 1.072, 1.298
Baseline Pre-diabetic (42 to <48 mmol/mol)	HbA1c (mmol/mol)	Absolute Change	-7.49 (-7.77, -7.22)	<0.001	<0.001	-7.49 (-7.77, -7.22)	<0.001	<0.001	0.684	1.000 (0.858, 2.068)	0.872, 0.920, 0.976, 1.048, 1.202
Baseline Normal (<42 mmol/mol)	HbA1c (mmol/mol)	Absolute Change	-2.38 (-2.48, -2.27)	<0.001	<0.001	-2.34 (-2.45, -2.24)	<0.001	<0.001	0.612	1.000 (0.903, 2.150)	0.917, 0.943, 0.970, 1.013, 1.192

IPW = inverse probability weighting. Complete-case estimates include participants with paired baseline and follow-up measurements for each outcome. IPW estimates reweighted participants with complete paired outcome data to represent the relevant target population. For the primary weight outcomes and overall secondary blood-test outcomes, the target population was the full analytical cohort of 8,127 participants. For analyses stratified by baseline HbA1c category, the target population was the corresponding baseline-defined HbA1c subgroup, as observed baseline HbA1c was required to classify participants as diabetic, pre-diabetic, or normal.

Stabilised inverse probability weights were estimated using prespecified baseline covariates, including age group, baseline BMI category, ethnicity, IMD quintile, and baseline HbA1c. Missing covariate values were handled using missing-indicator categories. The c-statistic describes the discrimination of the logistic regression model used to estimate each participant’s probability of having complete paired outcome data. Weight distributions were manually assessed to identify extreme weights and determine whether truncation was required. No truncation was necessary here. Benjamini-Hochberg false discovery rate (FDR) adjusted P values are presented alongside estimates for secondary outcomes to account for multiple testing. FDR adjustment was not applied to primary weight outcomes. FDR adjustment was applied separately within two groups of secondary outcomes: the six main blood-test outcomes and the three baseline HbA1c category-specific outcomes.

## Code availability

Analytical code for data processing and statistical analysis is available via GitHub at https://github.com/jamiewlc/shemed-glp1-6m-clinical-outcomes and Zenodo at https://doi.org/10.5281/zenodo.18821943 (ref. ^22^).
