# Supplementary material for: Real-world, digital-first delivery of GLP-1 receptor agonist therapy in women
Source: Nat Med. 2026 Oct 16;32(11):4319–29. doi: 10.1038/s41591-026-04687-x (PMC13768194; doi:10.1038/s41591-026-04687-x)
Supplement: Supplementary file 1 [file 41591_2026_4687_MOESM1_ESM.pdf]

nature medicine

Supplementary information https://doi.org/10.1038/s41591-026-04687-x

Real-world, digital-first delivery of GLP-1 receptor agonist therapy in women

In the format provided by the authors and unedited

SUPPLEMENTARY METHODS

Study design and setting

This was a real-world, observational cohort study of women enrolled in the SheMed GLP-1 programme, a digital-first, direct-to-consumer obesity treatment service delivered in the UK. The programme combines remote onboarding, on-demand Proctor-supported verification, supervised at-home capillary blood testing, app-based questionnaires, weekly video check-ins with trained Proctors, structured medication adherence verification, proactive side-effect mitigation and monthly clinical review to guide GLP-1 receptor agonist prescribing, refill approval and individualised dose titration.

The analysis included participants recruited between 27 November 2024 and 31 January 2025 who met predefined eligibility criteria, initiated GLP-1 receptor agonist therapy with tirzepatide or semaglutide, and had at least one follow-up assessment during the six-month observation window.

Participants

Potential participants were identified via social media and web advertising and directed to an online onboarding portal. Initial eligibility was assessed using a structured digital screening questionnaire capturing demographic characteristics, weight and height to calculate body mass index, medical history, current medications, weight history, lifestyle information and potential contraindications to GLP-1 receptor agonist therapy.

Eligibility and exclusion criteria

Inclusion criteria were:

- Female sex at birth
- Age ≥18 years
- BMI ≥30 kg/m²; or BMI ≥27 kg/m² with at least one weight-related comorbidity, including type 2 diabetes, prediabetes, hypertension, dyslipidaemia, asthma or COPD requiring regular inhaled corticosteroids, polycystic ovary syndrome, obstructive sleep apnoea, heart disease or other clinician-defined weight-related conditions; or BMI ≥27.5 kg/m² and belonging to an ethnic or racial group at higher risk for weight-related complications (South Asian, Chinese, Other Asian, Black African, African-Caribbean or Middle Eastern) consistent with NICE guidance.

Key exclusion criteria included age <18 or >80 years, personal or family history of medullary thyroid carcinoma or multiple endocrine neoplasia type 2, history of pancreatitis or pancreatobiliary malignancy, active gallbladder disease, severe liver disease or ALT/AST >95 U/L, severe hypertriglyceridaemia with triglycerides >10 mmol/L, severe renal impairment, significant thyroid dysfunction defined as TSH <0.1 or >30 mIU/L, uncontrolled cardiovascular disease, type 1 diabetes, uncontrolled type 2 diabetes with HbA1c >100 mmol/mol, current or recent cancer, inflammatory bowel disease, pregnancy or breastfeeding, current use of medications with a narrow therapeutic index or high-risk anticoagulation, and active substance misuse or severe psychiatric instability.

Individuals meeting initial eligibility criteria purchased an at-home capillary blood test kit (£49, refunded if subsequently ineligible). Participants were then invited to download the SheMed application and complete a live video onboarding session with a trained eMed Proctor. This session was available on demand and did not require appointment scheduling; participants were connected to a live Proctor through the application within approximately 30 seconds.

During the live Proctor session, participant identity was verified using photographic identification and a supervised baseline weigh-in was completed using the participant's own scales. Participants were instructed to use the same scales for subsequent weigh-ins throughout the programme to maximise consistency of longitudinal weight monitoring. During the same onboarding process, participants completed an additional detailed baseline questionnaire capturing full medical history, medication history, behavioural and lifestyle factors, life-stage information and other baseline health data.

Participants were then guided through capillary blood sample collection, packaging and return procedures during the supervised video session. Blood samples were processed by an accredited laboratory. The initial screening questionnaire, detailed baseline questionnaire, verified weight, laboratory results and any additional relevant clinical information, including NHS GP details, were then reviewed by the clinical team before a prescribing decision was made.

The SheMed digital programme

Following completion of the eligibility assessment, supervised onboarding and baseline blood testing, individuals whose clinical information was reviewed and approved by the clinical team were invited to join the SheMed programme. Joining the programme marked entry into a structured digital-first clinical pathway through which participants received GLP-1 receptor agonist treatment, ongoing monitoring, adherence support,

proactive side-effect mitigation and monthly clinical review. Individuals who were not considered clinically suitable did not progress to medication initiation through the programme.

All participants who joined the SheMed programme followed the same protocolised care pathway. The programme was designed to address several challenges associated with real-world GLP-1 receptor agonist treatment outside traditional clinic-based services, including medication non-adherence, inappropriate or automatic dose escalation, delayed recognition of adverse effects, unmanaged gastrointestinal symptoms and limited longitudinal clinical monitoring. A central and distinctive feature of the SheMed digital programme, in contrast to transactional digital prescribing models, was that ongoing participation was conditional on continued engagement with structured monitoring requirements. Participants were required to complete monthly questionnaires and undergo clinical review before medication refills were approved, meaning that continued access to treatment was linked to active participation, adherence monitoring and longitudinal safety oversight.

Participants deemed suitable for treatment were prescribed either tirzepatide or semaglutide according to clinician judgement and participant preference. Medication was dispensed and delivered directly to participants by post. Alongside medication, participants received a sharps bin and a structured side-effect support package containing over-the-counter medicines intended to support the early management of common gastrointestinal symptoms associated with GLP-1 receptor agonist therapy, including bloating, constipation, diarrhoea and reflux symptoms. This proactive side-effect mitigation programme was a distinctive component of the SheMed digital pathway and was designed to provide participants with immediate practical support at the point symptoms occurred, reduce symptom burden, improve treatment tolerability and support persistence with therapy.

Participants also had access through the application to an evolving library of educational content, including articles, videos and live webinars. Content covered injection technique, expected treatment effects, hydration, nutrition, side-effect expectations, side-effect self-management and programme details. These resources were reviewed and added to throughout participants' time on the programme, providing ongoing education and opportunities for engagement beyond scheduled Proctor and clinical review interactions. The educational content was intended to support medication initiation, reinforce safe self-administration, improve understanding of expected treatment and side-effect trajectories, and provide participants with information that could be accessed at the point of need throughout the programme.

A central component of the programme was weekly monitoring through live video check-ins with trained Proctors. Participants were able to join a live Proctor check-in session at least weekly through the SheMed application. These sessions provided a structured mechanism for adherence verification, weight monitoring, behavioural support and escalation of concerns. During check-ins, participants completed video-supported weigh-ins using the same scales as those used during onboarding, recorded injections, confirmed medication administration and documented the dose taken. Medication adherence was further supported through digital medication scanning and confirmation of dose use within the platform. These processes were designed to strengthen the reliability of real-world medication adherence and weight data.

The Proctor network also acted as an additional safety and support mechanism within the platform. Proctors did not provide clinical assessment or treatment advice, but could identify missed check-ins, possible non-adherence, participant questions, side effects or other concerns raised during sessions and escalate these on behalf of the participant to the appropriate clinical or non-clinical support team. Participants were also able to obtain support throughout the programme through Proctor interactions, digital support channels and escalation pathways within the application.

Participants were encouraged to complete at least three, and where possible four, weekly check-ins within each four-week cycle. Non-attendance at individual weekly check-ins did not result in penalties; however, automated reminders and app-based prompts supported ongoing engagement. Repeated non-engagement could be identified through the platform and escalated for further support where appropriate.

Participants could report side effects or clinical concerns at any time through the SheMed application using the side-effect reporting functionality, or by completing an online side-effect reporting form. When symptoms were reported through the application, participants were provided with immediate self-help advice within the app. When symptoms were reported through the online form, participants received immediate self-help advice by email. The side-effect support package provided with the first medication delivery was available for participants to use promptly to support symptom reduction where appropriate. Side-effect reports or clinical concerns could also be escalated to the clinical team, with an aim to review escalated concerns within one working day. Clinical review could result in tailored side-effect advice, dose modification, temporary treatment interruption, additional follow-up or escalation to urgent care pathways where clinically indicated.

Every four weeks, participants completed a structured refill questionnaire through the application. This captured updated health status, medication tolerability, side effects, adherence, weight trajectory, lifestyle information and any new clinical concerns. Completion of this questionnaire was required for continued participation in the programme and for medication refill approval. If a participant did not complete the questionnaire, automated reminders were sent through push notifications and email correspondence prompting completion. Participants who did not complete the required monthly questionnaire were unable to continue active participation in the programme or receive further medication through the programme until the review process had been completed and clinically approved.

Monthly clinical review was conducted using the refill questionnaire responses, weight trajectory, medication adherence data, side-effect information and any new clinical concerns. These reviews informed continuation of treatment, refill approval, individualised dosing decisions and escalation where required. Participants were also provided with opportunities to discuss treatment concerns or programme-related questions with clinicians during the review process.

In addition to weekly and monthly monitoring, participants completed detailed health, medical and lifestyle questionnaires through the SheMed application. These captured longitudinal data on weight, appetite, medication tolerability, side effects, physical activity, sleep, mood, lifestyle behaviours and women's health symptoms. Repeat at-home capillary blood testing was scheduled at six months to support longitudinal assessment of metabolic and safety markers. Six-month blood test kits were automatically sent to participants, and reminders were issued through the application and associated communication channels to prompt completion. The repeat blood testing process mirrored baseline procedures, including remote support for sample collection, packaging and return where required.

The programme was designed to be flexible and accessible while maintaining structured clinical oversight. There were no fixed clinic visits, and onboarding, monitoring, prescribing review, refill assessment, side-effect reporting and support interactions could be completed remotely. Participants had access to digital support, programme information, educational resources, webinars and escalation routes through the SheMed platform at the point of need.

Medication dosing and titration

Choice and dose of GLP-1 receptor agonist medication were determined by the clinical team at monthly reviews. Decisions were informed by weight trajectory, side-effect profile, medication adherence, refill questionnaire responses, participant preference and any new clinical concerns. Standard licensing guidance for dose escalation was followed, but dose escalation did not occur automatically.

Clinicians were advised not to escalate doses if participants demonstrated good weight loss, defined as ≥2 kg in the preceding month, or reported moderate or severe side effects. For tirzepatide and semaglutide, starting doses and incremental increases followed the relevant summary of product characteristics, with a focus on achieving the lowest effective dose for each individual. Participants could switch between agents for clinical reasons or supply constraints at the clinician's discretion.

Weight data cleaning and outlier management

Weight data were subjected to prespecified hierarchical cleaning procedures applied within individuals to identify implausible observations while preserving true biological variability. First, biologically implausible values were removed, including extreme entries inconsistent with human physiology (for example, values exceeding [500] kg). Second, potential lb–kg unit-entry errors were identified where recorded weight values were ≥1.8 times adjacent measurements. In these cases, values were converted by division by 2.2 when the resulting value lay within ±30% of the individual's median plausible weight. Third, remaining isolated deviations ≥30% relative to adjacent measurements were identified as outliers and removed. One-sided checks were applied at the beginning and end of individual trajectories where adjacent observations were unavailable. To verify algorithm performance, a random sample of participants with identified outliers underwent manual review. In addition, participants exhibiting extreme weight change between baseline and six months (≥99th percentile of weight loss or gain) were manually inspected for potential data-entry error, including comparison with proctored video measurements where available.

Laboratory data cleaning

Laboratory measurements were evaluated against prespecified biological plausibility thresholds. HbA1c values <10 mmol mol^{-1} or >150 mmol mol^{-1} were considered implausible and removed. Alanine aminotransferase (ALT) and aspartate aminotransferase (AST) values <5 or >500 U L^{-1} were excluded. All observed lipid measurements (total cholesterol, HDL cholesterol, LDL cholesterol and triglycerides) fell within biologically plausible ranges and were retained without modification.

Variable definitions

Because study participants resided across all four nations in the UK, socioeconomic status was derived from the most recent nation specific Index of Multiple Deprivation (IMD) based on participants' postcodes. Postcodes in England and Wales were mapped to 2021 Lower Layer Super Output Areas and linked to the 2025 English and Welsh IMD. Scottish postcodes were linked to 2011 Data Zones and mapped to the 2020 Scottish IMD. Northern Irish postcodes were mapped to 2001 Super Output Areas and linked to the 2017 Northern Ireland IMD. IMD quintiles were defined relative to the national distribution within each nation, as deprivation indices are constructed and ranked separately.

Glycaemic categories were defined using HbA1c thresholds corresponding to normoglycaemia (<42 mmol mol^{-1}), prediabetes (42–47 mmol mol^{-1}) and diabetes (≥48 mmol mol^{-1}). Treatment discontinuation was defined as cessation of pharmacotherapy during the six-month observation period, including participant withdrawal or absence of prescription renewal for >8 weeks within the expected prescribing interval of the programme. Adverse-effect severity was categorised using structured questionnaire responses as none, mild, moderate or severe. Participants were instructed to measure weight under standardised conditions (light clothing, no footwear where feasible) using personal weighing devices and to return capillary blood samples on the day of collection or the next working day using pre-paid priority postal packaging compliant with biological sample transport requirements.

Ethnicity and postcode-derived IMD were defined using the most complete available participant covariate extract prior to final analysis. Compared with the earlier submitted version of this manuscript, the resubmitted analyses used the final covariate extract, which included additional ethnicity and postcode information. Because participants could complete or update profile information after initial programme enrolment, and because the final extract incorporated a longer period for completion and updating of participant-submitted details, additional ethnicity and postcode information was available for a small number of participants. Where updated ethnicity or postcode information was available, these data were used to minimise missingness in baseline sociodemographic variables. These updates affected only the completeness of ethnicity and IMD variables, and did not alter participant eligibility, inclusion in the analytical cohort, baseline weight or BMI, outcome definitions, or the interpretation of the study findings.

Statistical modelling details

Linear regression models examining six month weight change across baseline subgroup variables, with nested model comparison performed using partial F-tests. Multivariable regression models evaluating associations between weight change and biomarker change included adjustment for age, ethnicity, IMD quintile and baseline weight category. Time-to-event analyses of treatment discontinuation were performed using Cox proportional hazards regression adjusted for age, ethnicity, IMD quintile and baseline weight category. Proportional hazards assumptions were assessed using Schoenfeld residuals, with no evidence that the assumption was violated. Subgroup Kaplan–Meier curves were presented for descriptive purposes without weighting. Forest-plot estimates representing biomarker change per 10 kg weight reduction were derived from adjusted linear regression models.

For secondary outcomes and exploratory analyses involving multiple related hypothesis tests, P values were adjusted using the Benjamini–Hochberg false discovery rate (FDR) procedure within prespecified groups of related comparisons presented within the same figure. FDR adjustment was not applied to the primary weight outcomes. For secondary biochemical outcomes, adjustment was applied across the six main blood-test outcome comparisons, comprising absolute HbA1c change, percentage HbA1c change, total cholesterol, HDL cholesterol, LDL cholesterol and triglycerides. A separate FDR adjustment was applied across the three HbA1c baseline-category analyses. For exploratory subgroup and supplementary analyses, FDR adjustment was applied within each figure or analysis specific group of tests, such as subgroup comparisons by baseline weight, age, IMD quintile and ethnicity.

Missing data

All main analyses were conducted using complete-case approaches for each endpoint including participants with paired baseline and follow-up measurements. Baseline sociodemographic and clinical characteristics were compared between participants with and without complete paired follow-up data to identify whether systematic differences existed between those included in and excluded from the analytical samples.

As a sensitivity analysis, primary and secondary paired change estimates were repeated using inverse probability weighting (IPW) to assess the potential influence of missing baseline or follow-up outcome data. For the primary weight and overall secondary blood test outcomes, the target population comprised the full analytical cohort of 8,127 participants. For subgroup-specific analyses, the target population comprised the relevant baseline-defined subgroup. In particular, analyses stratified by baseline HbA1c category were necessarily restricted to participants with an observed baseline HbA1c value, as baseline HbA1c was required to define diabetic, pre-diabetic, and normal HbA1c subgroups. Within each of these baseline HbA1c subgroups, IPW was used to reweight participants with complete paired outcome data to represent the corresponding baseline subgroup, rather than the full analytical cohort.

For each outcome, the probability of having complete paired baseline and follow-up measurements was estimated using logistic regression models. Models included age group, baseline BMI category, ethnicity, IMD quintile, and baseline HbA1c as explanatory variables. Missing covariate values were handled using missing-indicator categories. As missingness in key baseline covariates included as explanatory variables was low, as presented in Table 1, any additional bias introduced by this approach was expected to be minimal. Model discrimination was assessed using the c-statistic.

Stabilised inverse probability weights were calculated as the marginal probability of having complete paired outcome data divided by the predicted probability of having complete paired outcome data. Weight distributions were manually assessed to identify extreme weights and determine whether truncation was required. In this case, no truncation was necessary. Among participants with complete paired measurements, weighted mean within-person changes and corresponding confidence intervals were estimated using weighted intercept-only models, providing the IPW equivalent of the complete-case paired t-tests used in the main analyses.

Supplementary Table 1 | Baseline characteristics of the study population stratified by missingness in paired weight measurements

		Sample	
	Total (N=8127)	No Missingness (N=7297)	Missing Paired Weight (N=830)
**Sex**			
Female	8127 (100%)	7297 (100%)	830 (100%)
**Age**			
18 to <30	730 (9.0%)	572 (7.8%)	158 (19.0%)
30 to <40	2096 (25.8%)	1858 (25.5%)	238 (28.7%)
40 to <50	2468 (30.4%)	2281 (31.3%)	187 (22.5%)
50 to <60	2107 (25.9%)	1928 (26.4%)	179 (21.6%)
60 to <80	726 (8.9%)	658 (9.0%)	68 (8.2%)
**Weight (kg)**			
<90 kg	2108 (25.9%)	1828 (25.1%)	280 (33.7%)
90 to <100 kg	1771 (21.8%)	1591 (21.8%)	180 (21.7%)
100 to <110 kg	1495 (18.4%)	1365 (18.7%)	130 (15.7%)
110 to <120 kg	1183 (14.6%)	1076 (14.7%)	107 (12.9%)
≥120 kg	1570 (19.3%)	1437 (19.7%)	133 (16.0%)
**BMI (kg/m²)**			
Overweight (25.0 to <30.0)	369 (4.5%)	304 (4.2%)	65 (7.8%)
Obesity I (30.0 to <35.0)	2659 (32.7%)	2341 (32.1%)	318 (38.3%)
Obesity II (35.0 to <40.0)	2366 (29.1%)	2145 (29.4%)	221 (26.6%)
Obesity III (≥40.0)	2733 (33.6%)	2507 (34.4%)	226 (27.2%)
**Total Cholesterol (mmol/L)**			
Mean (SD)	5.71 (1.19)	5.73 (1.19)	5.57 (1.20)
Median (IQR)	5.59 (4.88, 6.43)	5.61 (4.90, 6.45)	5.46 (4.74, 6.26)
Range [Min, Max]	1.86, 12.9	2.29, 12.9	1.86, 10.5
Missing	594 (7.3%)	522 (7.2%)	72 (8.7%)
**HDL Cholesterol (mmol/L)**			
Mean (SD)	1.39 (0.365)	1.39 (0.364)	1.37 (0.377)
Median (IQR)	1.33 (1.13, 1.59)	1.33 (1.14, 1.59)	1.31 (1.10, 1.59)
Range [Min, Max]	0.300, 3.38	0.300, 3.38	0.590, 3.07
Missing	608 (7.5%)	539 (7.4%)	69 (8.3%)
**LDL Cholesterol (mmol/L)**			
Mean (SD)	3.77 (1.08)	3.78 (1.08)	3.65 (1.08)
Median (IQR)	3.67 (3.02, 4.42)	3.67 (3.02, 4.44)	3.60 (2.93, 4.25)
Range [Min, Max]	0.460, 10.2	0.460, 10.2	0.730, 8.40
Missing	623 (7.7%)	550 (7.5%)	73 (8.8%)
**Triglycerides (mmol/L)**			
Mean (SD)	1.64 (0.847)	1.64 (0.844)	1.59 (0.871)
Median (IQR)	1.45 (1.07, 1.97)	1.45 (1.07, 1.97)	1.40 (1.02, 1.94)
Range [Min, Max]	0.260, 10.4	0.260, 10.4	0.420, 7.50
Missing	636 (7.8%)	559 (7.7%)	77 (9.3%)
**HbA1c (mmol/mol)**			
Mean (SD)	36.2 (6.13)	36.2 (6.12)	35.9 (6.15)
Median (IQR)	35.7 (32.9, 39.0)	35.9 (33.0, 39.0)	35.3 (32.4, 38.6)
Range [Min, Max]	15.0, 111	15.0, 111	16.4, 91.6
Missing	319 (3.9%)	290 (4.0%)	29 (3.5%)
**Ethnicity**			
Non-White	942 (11.6%)	809 (11.1%)	133 (16.0%)
White	7154 (88.0%)	6463 (88.6%)	691 (83.3%)
Missing	31 (0.4%)	25 (0.3%)	6 (0.7%)
**Index of Multiple Deprivation (Quintile)**			
1 (Most Deprived)	1354 (16.7%)	1188 (16.3%)	166 (20.0%)
2	1695 (20.9%)	1526 (20.9%)	169 (20.4%)
3	1753 (21.6%)	1587 (21.7%)	166 (20.0%)
4	1792 (22.1%)	1629 (22.3%)	163 (19.6%)
5 (Least Deprived)	1531 (18.8%)	1365 (18.7%)	166 (20.0%)
Missing	2 (0.0%)	2 (0.0%)	0 (0%)

Baseline demographic, anthropometric and biochemical characteristics of participants initiating GLP-1 receptor agonist therapy, presented overall and stratified by whether missingness is present within paired weight measurements (i.e. absence of a complete paired baseline and 6 month follow-up measurement). Continuous variables are summarised as mean (standard deviation) and median (interquartile range), with ranges provided where relevant. Categorical variables are presented

as counts and percentages within each column. Missing values are reported for each variable. Body mass index (BMI) and HbA1c categories were defined according to standard clinical thresholds. Socioeconomic status was determined using nation-specific Indices of Multiple Deprivation (IMD) for England, Scotland, Wales and Northern Ireland, derived from participant postcodes. Biochemical measurements were obtained from capillary blood samples processed at an accredited laboratory. HDL, high-density lipoprotein; LDL, low-density lipoprotein; HbA1c, glycated haemoglobin; IMD, Index of Multiple Deprivation.

Supplementary Table 2 | Baseline characteristics of the study population stratified by missingness in paired blood test measurements

		Sample	
	Total (N=8127)	No Missingness (N=5282)	Any Missingness in Blood Test Measures (N=2845)
**Sex**			
Female	8127 (100%)	5282 (100%)	2845 (100%)
**Age**			
18 to <30	730 (9.0%)	406 (7.7%)	324 (11.4%)
30 to <40	2096 (25.8%)	1409 (26.7%)	687 (24.1%)
40 to <50	2468 (30.4%)	1728 (32.7%)	740 (26.0%)
50 to <60	2107 (25.9%)	1328 (25.1%)	779 (27.4%)
60 to <80	726 (8.9%)	411 (7.8%)	315 (11.1%)
**Weight (kg)**			
<90 kg	2108 (25.9%)	1277 (24.2%)	831 (29.2%)
90 to <100 kg	1771 (21.8%)	1130 (21.4%)	641 (22.5%)
100 to <110 kg	1495 (18.4%)	1010 (19.1%)	485 (17.0%)
110 to <120 kg	1183 (14.6%)	783 (14.8%)	400 (14.1%)
≥120 kg	1570 (19.3%)	1082 (20.5%)	488 (17.2%)
**BMI (kg/m²)**			
Overweight (25.0 to <30.0)	369 (4.5%)	198 (3.7%)	171 (6.0%)
Obesity I (30.0 to <35.0)	2659 (32.7%)	1671 (31.6%)	988 (34.7%)
Obesity II (35.0 to <40.0)	2366 (29.1%)	1561 (29.6%)	805 (28.3%)
Obesity III (≥40.0)	2733 (33.6%)	1852 (35.1%)	881 (31.0%)
**Total Cholesterol (mmol/L)**			
Mean (SD)	5.71 (1.19)	5.71 (1.16)	5.72 (1.28)
Median (IQR)	5.59 (4.88, 6.43)	5.61 (4.89, 6.42)	5.57 (4.84, 6.43)
Range [Min, Max]	1.86, 12.9	2.29, 11.8	1.86, 12.9
Missing	594 (7.3%)	0 (0%)	594 (20.9%)
**HDL Cholesterol (mmol/L)**			
Mean (SD)	1.39 (0.365)	1.39 (0.364)	1.40 (0.368)
Median (IQR)	1.33 (1.13, 1.59)	1.33 (1.13, 1.58)	1.34 (1.13, 1.61)
Range [Min, Max]	0.300, 3.38	0.300, 3.38	0.590, 3.25
Missing	608 (7.5%)	0 (0%)	608 (21.4%)
**LDL Cholesterol (mmol/L)**			
Mean (SD)	3.77 (1.08)	3.76 (1.05)	3.77 (1.16)
Median (IQR)	3.67 (3.02, 4.42)	3.67 (3.03, 4.41)	3.66 (2.97, 4.42)
Range [Min, Max]	0.460, 10.2	0.460, 10.2	0.730, 10.1
Missing	623 (7.7%)	0 (0%)	623 (21.9%)
**Triglycerides (mmol/L)**			
Mean (SD)	1.64 (0.847)	1.64 (0.838)	1.64 (0.869)
Median (IQR)	1.45 (1.07, 1.97)	1.45 (1.07, 1.96)	1.45 (1.05, 1.98)
Range [Min, Max]	0.260, 10.4	0.260, 10.4	0.420, 7.71
Missing	636 (7.8%)	0 (0%)	636 (22.4%)
**HbA1c (mmol/mol)**			
Mean (SD)	36.2 (6.13)	36.2 (6.13)	36.2 (6.12)
Median (IQR)	35.7 (32.9, 39.0)	35.7 (32.9, 38.9)	36.0 (32.8, 39.1)
Range [Min, Max]	15.0, 111	15.0, 111	16.3, 91.6
Missing	319 (3.9%)	0 (0%)	319 (11.2%)
**Ethnicity**			
Non-White	942 (11.6%)	512 (9.7%)	430 (15.1%)
White	7154 (88.0%)	4757 (90.1%)	2397 (84.3%)
Missing	31 (0.4%)	13 (0.2%)	18 (0.6%)
**Index of Multiple Deprivation (Quintile)**			
1 (Most Deprived)	1354 (16.7%)	838 (15.9%)	516 (18.1%)
2	1695 (20.9%)	1084 (20.5%)	611 (21.5%)
3	1753 (21.6%)	1153 (21.8%)	600 (21.1%)
4	1792 (22.1%)	1200 (22.7%)	592 (20.8%)
5 (Least Deprived)	1531 (18.8%)	1006 (19.0%)	525 (18.5%)
Missing	2 (0.0%)	1 (0.0%)	1 (0.0%)

Baseline demographic, anthropometric and biochemical characteristics of participants initiating GLP-1 receptor agonist therapy, presented overall and stratified by whether missingness is present within any paired blood test measurements (i.e. absence of complete paired baseline and 6 month follow-up measurements across any of the following measures: total cholesterol, HDL cholesterol, LDL cholesterol, triglycerides, HbA1c). Continuous variables are summarised as mean (standard deviation) and median (interquartile range), with ranges provided where relevant. Categorical variables are presented as counts and percentages within each column. Missing values are reported for each variable. Body mass index (BMI) and HbA1c categories were defined according to standard clinical thresholds. Socioeconomic status was determined using nation-specific Indices of Multiple Deprivation (IMD) for England, Scotland, Wales and Northern Ireland, derived from participant postcodes. Biochemical measurements were obtained from capillary blood samples processed at an accredited laboratory. HDL, high-density lipoprotein; LDL, low-density lipoprotein; HbA1c, glycated haemoglobin; IMD, Index of Multiple Deprivation.
