# Supplementary material for: Real-world, digital-first delivery of GLP-1 receptor agonist therapy in women
Source: Nat Med. 2026 Oct 16;32(11):4319–29. doi: 10.1038/s41591-026-04687-x (PMC13768194; doi:10.1038/s41591-026-04687-x)
Supplement: Supplementary file 2 [file 41591_2026_4687_MOESM2_ESM.pdf]

Corresponding author(s): John Deanfield

Last updated by author(s): 2026/07/28

Reporting Summary

Nature Portfolio wishes to improve the reproducibility of the work that we publish. This form provides structure for consistency and transparency in reporting. For further information on Nature Portfolio policies, see our Editorial Policies and the Editorial Policy Checklist.

Statistics

For all statistical analyses, confirm that the following items are present in the figure legend, table legend, main text, or Methods section.

n/a	Confirmed	
☐	☒	The exact sample size (*n*) for each experimental group/condition, given as a discrete number and unit of measurement
☐	☒	A statement on whether measurements were taken from distinct samples or whether the same sample was measured repeatedly
☐	☒	The statistical test(s) used AND whether they are one- or two-sided<br>*Only common tests should be described solely by name; describe more complex techniques in the Methods section.*
☐	☒	A description of all covariates tested
☐	☒	A description of any assumptions or corrections, such as tests of normality and adjustment for multiple comparisons
☐	☒	A full description of the statistical parameters including central tendency (e.g. means) or other basic estimates (e.g. regression coefficient) AND variation (e.g. standard deviation) or associated estimates of uncertainty (e.g. confidence intervals)
☐	☒	For null hypothesis testing, the test statistic (e.g. *F*, *t*, *r*) with confidence intervals, effect sizes, degrees of freedom and *P* value noted<br>*Give P values as exact values whenever suitable.*
☒	☐	For Bayesian analysis, information on the choice of priors and Markov chain Monte Carlo settings
☒	☐	For hierarchical and complex designs, identification of the appropriate level for tests and full reporting of outcomes
☐	☒	Estimates of effect sizes (e.g. Cohen's *d*, Pearson's *r*), indicating how they were calculated

Our web collection on statistics for biologists contains articles on many of the points above.

Software and code

Policy information about availability of computer code

Data collection	Data were collected through the SheMed secure digital platform and smartphone application. Data sources included structured online onboarding and refill questionnaires, participant reported demographic and medical information, remotely recorded weight measurements, live video supported identity and weight verification, medication scanning and adherence records, side-effect reports, prescription and dispensing records, and baseline and six month capillary blood test results. Live video onboarding and monitoring were supported by trained eMed Proctors. Laboratory samples were processed by Randox Clinical Laboratory Service, a UKAS accredited laboratory.<br><br>The supplementary methods describe weekly video supported weigh-ins, medication scanning and monthly refill questionnaires as central components of data collection as part of the SheMed programme.
Data analysis	All data processing, statistical analysis and visualisation were performed using R version 4.5.2. Analytical code for data processing and statistical analysis is available through GitHub and archived on Zenodo at DOI: 10.5281/zenodo.18821943

For manuscripts utilizing custom algorithms or software that are central to the research but not yet described in published literature, software must be made available to editors and reviewers. We strongly encourage code deposition in a community repository (e.g. GitHub). See the Nature Portfolio guidelines for submitting code & software for further information.

Data

Policy information about availability of data

All manuscripts must include a data availability statement. This statement should provide the following information, where applicable:

- Accession codes, unique identifiers, or web links for publicly available datasets
- A description of any restrictions on data availability
- For clinical datasets or third party data, please ensure that the statement adheres to our policy

Data and an accompanying data dictionary will be available for five years from publication to qualified researchers affiliated with recognized institutions, subject to applicable regulatory requirements and institutional approval. Requests should include the research objectives, methods, details of ethical approval and required data and be submitted to support@shemed.co.uk. Requests will be assessed within six months for scientific validity. Approved applicants will receive de-identified participant level data after signing a data sharing agreement covering data security, permitted use and onward sharing.

Research involving human participants, their data, or biological material

Policy information about studies with human participants or human data. See also policy information about sex, gender (identity/presentation), and sexual orientation and race, ethnicity and racism.

Reporting on sex and gender

The programme was designed specifically for adult women, with eligibility defined according to self-reported female sex at birth during structured onboarding. This focus was intended to address the limited female specific implementation evidence for GLP-1RA treatment and the underrepresentation of women in large cardiometabolic outcome trials in obesity. The reported analyses were therefore restricted to participants who were female at birth. Because all 8,127 included participants were female at birth, comparisons between sexes were not applicable to the prespecified research question.

Reporting on race, ethnicity, or other socially relevant groupings

Ethnicity was self-reported during programme onboarding using standard UK census-style categories. Owing to the limited number of participants in individual minority ethnic groups, ethnicity was categorised as White or non-White for subgroup and adjusted analyses.

Socioeconomic status was derived using area-level Index of Multiple Deprivation measures derived from self-reported residential postcodes. Because participants lived across all four UK nations, postcodes were linked to the relevant nation-specific deprivation index, and participants were classified into national deprivation quintiles. Quintile 1 represented the most deprived areas and quintile 5 the least deprived areas.

Ethnicity and deprivation were included as covariates in adjusted analyses of treatment discontinuation and biomarker outcomes. They were also examined in subgroup analyses of weight outcomes.

Population characteristics

The analytic cohort comprised 8,127 adults who were female at birth and initiated GLP-1 receptor agonist treatment through the SheMed digital programme in the United Kingdom. The cohort spanned a wide adult age range (18–79 years), with 82.1% aged 30 to <60 years. Mean baseline weight was 104.2 kg and mean baseline BMI was 38.2 $kg m^{-2}$. Approximately 95.5% met criteria for class I-III obesity. Participants were predominantly White, although participants from multiple ethnic groups were included. Participants lived across England, Scotland, Wales and Northern Ireland and represented all national Index of Multiple Deprivation quintiles. The programme was a direct-access, partly subsidised, self-pay service; therefore, the cohort may not be representative of all people eligible for GLP-1RA treatment, and is noted as a limitation in the manuscript.

Eligibility criteria included female sex at birth and age 18–80 years and body mass index (BMI) ≥30 $kg m^{-2}$, or BMI 27–30 $kg m^{-2}$ with a qualifying weight-related comorbidity (including type 2 diabetes, prediabetes, hypertension, dyslipidaemia, asthma/COPD on regular inhaled corticosteroids, polycystic ovary syndrome, obstructive sleep apnoea and heart disease) or ethnicity-specific BMI threshold consistent with NICE guidance (South Asian, Chinese, Other Asian, Black African, African-Caribbean or Middle Eastern). Exclusion criteria comprised GLP-1RA use within the preceding 12 months, contraindicated medical conditions or medications, and pregnancy or breastfeeding.

Recruitment

Potential participants were recruited through social media and web advertising and directed to the SheMed online onboarding portal. Recruitment occurred between 27 November 2024 and 31 January 2025. Participants self-enrolled in the direct-to-consumer programme and paid a fixed programme fee covering medication, laboratory testing, digital monitoring and clinical support. The programme was partly subsidised by the provider during the study period.

Initial screening was conducted using a structured online questionnaire. Potentially eligible individuals then completed identity verification, supervised baseline weight confirmation, questionnaires and at-home capillary blood testing. Clinical information and laboratory results were reviewed before treatment was prescribed. Participants prescribed and dispatched GLP-1RA medication before the administrative cut-off and initiating treatment by 9 February 2025 formed the analytic cohort.

Potential sources of selection bias include self-selection in response to digital advertising, ability and willingness to use a smartphone based service, willingness and ability to pay programme costs, completion of baseline blood testing and onboarding, clinical exclusion after laboratory review, and the restriction to people seeking treatment through a female-focused digital programme. Of the initially screened population, 4,025 otherwise eligible individuals were not included because they did not complete baseline blood testing, and a further 308 were excluded following clinical review. These processes may limit representativeness and external generalisability.

Ethics oversight

Participants provided electronic consent during programme onboarding for use of their data for service evaluation and research purposes. Data were pseudonymised prior to transfer for analysis. In accordance with UK Health Research Authority

guidance, the study constituted secondary analysis of routinely collected service data and did not require research ethics committee review. The study was conducted in accordance with the Declaration of Helsinki and applicable data-protection legislation.

Note that full information on the approval of the study protocol must also be provided in the manuscript.

Field-specific reporting

Please select the one below that is the best fit for your research. If you are not sure, read the appropriate sections before making your selection.

☒ Life sciences ☐ Behavioural & social sciences ☐ Ecological, evolutionary & environmental sciences

For a reference copy of the document with all sections, see nature.com/documents/nr-reporting-summary-flat.pdf

Life sciences study design

All studies must disclose on these points even when the disclosure is negative.

Sample size

No formal a priori sample size calculation was performed. This was a retrospective analysis of routinely collected data from a real-world digital treatment programme. The sample comprised all participants who met the eligibility criteria, completed the required baseline assessments, received clinical approval, and were prescribed and dispatched GLP-1RA medication before the administrative cut-off, with treatment initiation by 9 February 2025. The resulting analytic cohort included 8,127 participants.

The available cohort was considered sufficient to estimate the primary within-participant change in weight with high precision and to examine secondary biochemical, safety and subgroup outcomes. Sample sizes for individual analyses were determined by the availability of the relevant baseline and follow-up measurements and are reported for each endpoint.

Data exclusions

Participant level eligibility exclusions were defined before analysis and were applied through the programme's clinical screening and prescribing pathway. Individuals were excluded if they did not complete the required baseline blood test or supervised onboarding, did not have a verified baseline weight, had contraindications or abnormal laboratory findings, were not clinically approved, or did not receive GLP-1RA medication before the administrative cut-off.

For analysis, weight data were subjected to prespecified hierarchical cleaning procedures applied within individuals to identify implausible observations while preserving true biological variability. First, biologically implausible values were removed, including extreme entries inconsistent with human physiology (for example, values exceeding 500 kg). Second, potential lb–kg unit-entry errors were identified where recorded weight values were ≥1.8 times adjacent measurements. In these cases, values were converted by division by 2.2 when the resulting value lay within ±30% of the individual's median plausible weight. Third, remaining isolated deviations ≥30% relative to adjacent measurements were identified as outliers and removed. One-sided checks were applied at the beginning and end of individual trajectories where adjacent observations were unavailable. To verify algorithm performance, a random sample of participants with identified outliers underwent manual review. In addition, participants exhibiting extreme weight change between baseline and six months (≥99th percentile of weight loss or gain) were manually inspected for potential data-entry error, including comparison with proctored video measurements where available.

Laboratory measurements were evaluated against prespecified biological plausibility thresholds. HbA1c values <10 mmol mol^{-1} or >150 mmol mol^{-1} were considered implausible and removed. Alanine aminotransferase (ALT) and aspartate aminotransferase (AST) values <5 or >500 U L^{-1} were excluded. All observed lipid measurements (total cholesterol, HDL cholesterol, LDL cholesterol and triglycerides) fell within biologically plausible ranges and were retained without modification.

Replication

Formal experimental replication was not applicable because this was an observational analysis of a real-world cohort rather than a laboratory or interventional experiment. Reproducibility was supported by making the analytical code for data processing, analysis and visualisation publicly available.

Randomization

Not applicable, as participants were not randomised to treatment or programme components. GLP-1RA selection and dose were determined by treating clinicians according to licensed prescribing guidance, clinical judgement, participant preference, treatment response and tolerability. Where relevant, multivariable regression analyses included mutual adjustment for age, ethnicity, Index of Multiple Deprivation quintile and baseline weight category. However, residual and unmeasured confounding remain possible because of the observational design.

Blinding

Blinding was not applicable to programme participants or treating clinicians because medication was prescribed and delivered as part of routine clinical care. Participants, proctors and clinicians were aware of treatment and dose. The independent statistical analysis team received pseudonymised programme data, but the analysis was not described as blinded to treatment, exposure or outcome values, and treatment identity was required for dose and treatment analyses.

Reporting for specific materials, systems and methods

We require information from authors about some types of materials, experimental systems and methods used in many studies. Here, indicate whether each material, system or method listed is relevant to your study. If you are not sure if a list item applies to your research, read the appropriate section before selecting a response.

Materials & experimental systems

n/a	Involved in the study
☒	☐ Antibodies
☒	☐ Eukaryotic cell lines
☒	☐ Palaeontology and archaeology
☒	☐ Animals and other organisms
☐	☒ Clinical data
☒	☐ Dual use research of concern
☒	☐ Plants

Methods

n/a	Involved in the study
☒	☐ ChIP-seq
☒	☐ Flow cytometry
☒	☐ MRI-based neuroimaging

Clinical data

Policy information about clinical studies

All manuscripts should comply with the ICMJE guidelines for publication of clinical research and a completed CONSORT checklist must be included with all submissions.

Clinical trial registration

Not applicable, as this was a retrospective observational analysis of routinely collected data from a clinical service and was not a prospective interventional clinical trial. Participants were not randomised, and the analysis did not assign an intervention under a research protocol.

Study protocol

A formal prospective trial protocol was not produced because the programme operated as a routine digital clinical service and the study was a retrospective observational analysis of routinely collected service data. The programme's eligibility criteria, clinical pathway, monitoring procedures, outcome definitions, data cleaning rules and statistical methods are described in the manuscript and Supplementary Methods. Analytical code is publicly archived through GitHub and Zenodo.

Data collection

Participants were recruited between 27 November 2024 and 31 January 2025 across the United Kingdom, and initiated GLP-1RA treatment by 9 February 2025. Follow-up continued for six months from each participant's treatment-initiation date.

Baseline data were collected during structured online onboarding and included demographic characteristics, sex, ethnicity, postcode, self-reported height, medical history, medication use, weight history and eligibility information. Baseline weight was recorded using participant owned digital scales and confirmed during supervised video onboarding. Baseline capillary blood samples were collected at home under remote guidance and processed by a UKAS-accredited laboratory.

During follow-up, weight measurements were prompted weekly and were supported through live video check-ins. Monthly refill questionnaires captured medication use, dose, adherence, side effects, wellbeing, weight trajectory, pregnancy status, new clinical concerns and concomitant medication use. Prescription and dispensing records were collected through the programme platform. Participants could report side effects at any time through the application or an online reporting route. Repeat capillary blood testing was scheduled at six months and assessed HbA1c and lipid concentrations.

Outcomes

The primary effectiveness endpoint was change in body weight from baseline to six months following treatment initiation, expressed as both absolute (kg) and percentage change. Secondary effectiveness endpoints included the proportion of participants achieving clinically meaningful weight-loss thresholds (≥5%, ≥10%, ≥15% and ≥20% of baseline weight) and changes in metabolic parameters over the same period. Glycaemic outcomes were assessed using HbA1c measured at baseline and six months, with transitions between normoglycaemia ($<42\,\text{mmol}\,\text{mol}^{-1}$), prediabetes (42 to $<48\,\text{mmol}\,\text{mol}^{-1}$) and diabetes ($\geq 48\,\text{mmol}\,\text{mol}^{-1}$) evaluated. Lipid outcomes included within-participant changes in total cholesterol, LDL cholesterol, HDL cholesterol and triglycerides. Programme engagement and tolerability were assessed through treatment discontinuation during follow-up and participant-reported side effects. Treatment discontinuation was defined as absence of a recorded GLP-1RA dispensing event for >8 weeks following the most recent dispense. Safety outcomes included frequency and severity of reported side effects during the observation period.

Plants

Seed stocks

Not applicable, as plants were not involved in this study.

Novel plant genotypes

Not applicable, as plants were not involved in this study.

Authentication

Not applicable, as plants were not involved in this study.
