# Supplementary material for: Real-world, digital-first delivery of GLP-1 receptor agonist therapy in women
Source: Nat Med. 2026 Oct 16;32(11):4319–29. doi: 10.1038/s41591-026-04687-x (PMC13768194; doi:10.1038/s41591-026-04687-x)
Supplement: Supplementary file 3 [file 41591_2026_4687_MOESM3_ESM.pdf]

nature portfolio

Peer Review File

Real world, digital-first delivery of GLP-1 receptor agonist therapy in women

Corresponding Author: Professor John Deanfield

This file contains all reviewer reports in order by version, followed by all author rebuttals in order by version.

Version 0:

Reviewer comments:

Reviewer #1

(Remarks to the Author)
Summary of Key Results: The authors report six-month outcomes in 8,127 women receiving GLP-1 receptor agonist therapy (almost entirely tirzepatide) via a fully digital, self-pay care model in the UK. Mean weight loss was 16.1 kg (15.5%), with high proportions achieving ≥10% (83%) and ≥20% (22%) weight loss. Modest but statistically significant improvements in HbA1c and lipid parameters were observed among those with paired data. Retention at six months was reported as 91%, and no serious treatment-related adverse events were identified.

Originality and Significance: The scale of the cohort and the exclusive focus on women are notable and address a genuine gap in the GLP-1 literature, which is dominated by male-predominant RCTs. However, the study is not methodologically novel. Similar real-world observational cohorts evaluating GLP-1RAs (including digital or routine-care pathways) have already been published. The principal contribution is therefore descriptive and implementation-focused, rather than hypothesis-testing or mechanistic.

Significance is moderate, although would benefit from reframing as real-world implementation evidence rather than effectiveness evidence.

Data & Methodology
Validity of Approach: The observational cohort design is appropriate for service evaluation but cannot support causal claims about treatment effectiveness or equivalence to randomized trials. The absence of a comparator group (usual care, non-digital care, or lifestyle-only) substantially limits interpretability. The self-pay, self-enrolled model with subsidization during the study period introduces strong selection bias that the authors acknowledged. The fact that nearly half of the screened population was excluded might warrant comparison of baseline characteristics (which understandably may not be possible).

Quality of Data: Weight was measured using participant-owned scales and height was self-reported. While pragmatic, these data sources introduce measurement error that is not formally quantified or analyzed. Approximately 20% of participants lacked paired biochemical data and ~10% lacked 6-month weight data.

Quality of Presentation: Tables and figures are generally clear and comprehensive.

Appropriate Use of Statistics and Treatment of Uncertainty: Statistical methods (paired t-tests, linear regression, Cox models) are standard and generally applied correctly.
All analyses are complete-case only, with no multiple imputation or inverse probability weighting.
Subgroup analyses are exploratory but not corrected for multiplicity, increasing the risk of false-positive interpretation.
Confidence intervals are reported appropriately, but uncertainty related to selection bias and missing data is underplayed.

Conclusions: The central descriptive finding—substantial short-term weight loss among retained participants—is internally consistent.
However, conclusions frequently overreach, implying effectiveness “comparable to trials” and equity across socioeconomic strata without sufficient methodological support.
Claims regarding safety, generalisability, and health equity are not robust given short follow-up, self-reporting, and selective inclusion.

Suggested Improvements for Possible Revision:
Reframe the manuscript explicitly as descriptive implementation research, avoiding causal or equivalence language.
Provide a systematic comparison of included vs excluded participants, particularly those excluded due to incomplete labs.
Add a clear limitations box in the main text highlighting selection bias, measurement error, and commercial context.

References and Prior Work: Key trials and recent real-world studies are cited appropriately.
However, prior digital-health GLP-1 observational studies and direct-to-consumer critiques should be more explicitly discussed in the Introduction to contextualize novelty and limitations.

Clarity and Context: Overall good clarity, but interpretive restraint is insufficient.

Final Recommendation
Reject (with encouragement to resubmit after major revision).
Rationale:
While the dataset is large and of clear interest, the study's internal validity limitations, high risk of selection bias, complete-case analytic strategy, and overinterpretation of results preclude acceptance in its current form as original clinical research. With substantial reframing, methodological strengthening, and more cautious conclusions, the work may be suitable for a digital health or implementation science journal.

Reviewer #2

(Remarks to the Author)
A. Summary of the key results
This manuscript presents an observational study evaluating outcomes from a digitally delivered weight-loss programme among women living with overweight or obesity. Most participants used Tirzepatide and weight outcomes were self-reported. The authors report a mean weight reduction of 15.5% from baseline, broadly consistent with results from recent clinical trials. Improvements were also observed in metabolic parameters, including HbA1c and lipid profiles. Participant retention was high, with 91.4% remaining in the programme at six months.

B. Originality and significance
The study provides useful real-world data on a digital weight-management intervention in combination with mainly Tirzepatide therapy. While the overall findings align with published clinical trial results, the real-world setting and digital-first model add valuable context. The authors should ensure comparison with relevant prior literature evaluating GLP-1RA use in digital or remote-care settings.

C. Data and methodology
The overall study design, analysis and presentation are clear. However, the absence of explicit exclusion criteria should be addressed. Participant characteristics indicate a predominantly healthy cohort with limited comorbidity, which restricts generalisability to broader populations who more commonly require GLP-1RA therapy due to obesity-related complications. The reliance on self-reported weight data is a limitation and warrants further discussion.

D. Appropriate use of statistics and treatment of uncertainties
Statistical methods appear appropriate, though further detail on handling of missing data, verification of self-reported outcomes and uncertainty estimates would strengthen the analysis.

E. Conclusions
The conclusions are broadly supported by the results, but the authors should temper statements regarding generalisability given the highly selected, largely white, comparatively healthy study population. The robustness of the findings is also limited by self-reporting and the lack of clarity regarding the nature and frequency of clinical follow-up.

F. Suggested improvements
• Provide a clear statement of inclusion and exclusion criteria.
• Clarify whether participants received regular clinical follow-up, physician or dietitian consultation, or wraparound care.
• Discuss whether programme fees affected uptake or demographic skew, particularly the underrepresentation of underserved communities.
• Describe any mechanisms used to validate self-reported outcomes or, if none, more fully acknowledge this limitation.
• Explain the absence of a 12-month follow-up period. Weight trajectories with GLP-1RA therapies often peak or plateau around 12 months and omission of this time point limits understanding of maximal effect and durability.

G. References
The reference list appears appropriate and credits relevant prior work.

H. Clarity and context
The manuscript is clearly written, and the abstract, introduction and conclusions are generally well-structured. Strengthening the discussion of generalisability, limitations and follow-up duration would improve contextual clarity.

Reviewer #3

(Remarks to the Author)
Deanfield and colleagues have submitted a well written manuscript on the outcomes of a digitally delivered program for over 8000 women based in the UK, where incretin therapies (mainly Tirzepatide) were prescribed and managed completely remotely including capillary blood tests being sent out, self weighing and completion of questionnaires and reporting of side effects. This was supported by a clinical and non-clinical team as well as an app, and outcomes were assessed at 6 months. Mean dose of Tirzepatide was 9.5mg at study end and mean weight loss was 15.5% of baseline weight with reductions in mean HbA1c and improvements in lipid profile, where around 90% provided weight and 80% completed biochemical assessment at 6 months.

The use of a digital only platform to prescribe and monitor incretin therapy is not entirely novel, as there are multiple telehealth providers that are doing this in various countries. However, targeting only adult women and being monitored and supported by an app and a clinical and non-clinical team being available to help deal with side effects or queries does make this different. The trial seemed to recruit over 8000 women in around 2 months, which is quite impressive. The data are clearly presented and the use of video or photos to ensure reported weight is correct adds validity to the self reported weight. Lack of data on blood pressure is noted, especially when discussing cardiometabolic risk. Follow up at 6 months are not long enough to determine long term outcomes but does suggest this is potentially a feasible long term model of care.

The methods describe the digitally delivered program with monthly follow up. There is brief mention that this was a program that required a fixed fee that was partially subsidised. Would the authors be able to expand on what amount was paid, and how this compares to simply buying the medication with a prescription from their primary care physician? There is an inherent selection bias in such a program that includes people who can afford to self pay, as well as in terms of willingness to pay and be involved in this study. Cost of the program itself in addition to the cost to the participant is also a major factor in determining whether or not such a program is scalable and sustainable.

While pregnancy was excluded at baseline, was it also discussed in the interim visits alongside contraception? It would be more important in a woman only study, given that these medications are not safe in pregnancy. Were there any pregnancies reported in the study duration?
There is a lack of detail of medications being taken, particularly in Type 2 diabetes and dyslipidaemia as the outcomes include HbA1c and lipid profiles. Were there any changes in medications during the study, and were there any additional (or fewer) medications taken that may have influenced these outcomes?
Was there a specific rationale behind use of >2kg/month as a reason not to uptitrate medication, as this was not used in the clinical trials, and the baseline weight/BMI of patients in this study was 38.2. So presumably greater weight loss may have been beneficial, as long as tolerance was not a problem?
For HbA1c, in addition to mmol/mol, it would be helpful to also have % values.
In Table 5, perhaps it is better to use Tirzepatide rather than Mounjaro to be consistent with the rest of the manuscript.

Version 1:

Reviewer comments:

Reviewer #1

(Remarks to the Author)
1. Summary of key results
This is a real-world observational service evaluation of 8,127 women initiating GLP-1RA therapy (99.8% tirzepatide) through a UK digital programme, with six-month follow-up. Key findings: mean weight loss of 16.1 kg (15.5%), with 83.1% achieving ≥10% loss; substantial HbA1c and lipid improvements; 91.4% retention; no serious treatment-related adverse events.

2. Originality and significance
The exclusive focus on women is a genuine and useful contribution. The authors' response letter reframes the contribution as "implementation evidence" rather than effectiveness evidence, which I agree is the more defensible framing and should be reflected more consistently in the abstract. The novelty rests on the bundle of monitoring components (mandatory labs, weekly video check-ins, structured titration) in my opinion.

3. Data & methodology: validity of approach, IPW weighting for missingness
The authors describe stabilised IPW models for probability of complete paired outcome data, using age group, baseline BMI category, ethnicity, IMD quintile, and baseline HbA1c as predictors. This approach can only correct for missingness that is missing at random (MAR) conditional on these observed covariates. It cannot correct for missing-not-at-random (MNAR) mechanisms, which are highly plausible here. I think it's a bit much to try and do more sensitivity analyses for MNAR because I don't think the overall message will change, but something to consider.

The rebuttal states IPW-weighted estimates were "materially similar" to complete-case estimates. This is presented as reassurance, but if the missingness model has limited discriminative power (i.e., the included covariates weakly predict missingness), IPW and complete-case estimates would converge. The authors should report a measure of the missingness model's discriminatory ability (e.g., c-statistic) if they haven't. I wasn't able to find the supplementary methods.

4. Appropriate use of statistics and treatment of uncertainties
It seems like hazard ratios for IMD quintiles 2 and 5 not differing significantly from quintile 1, while quintiles 3–4 do, is an unusual, non-monotonic pattern that is not discussed — this should be commented on as it slightly undercuts the no

meaningful socioeconomic gradient narrative.

5. Conclusions: robustness, validity, reliability
No additional comments.

6. Suggested improvements
Report the IPW weight distribution and missingness-model discrimination statistic (as above).
Discuss the non-monotonic IMD gradient in discontinuation hazard.

7. References
8. Clarity and context
The manuscript is clearly written and well-organised. The abstract could be strengthened by including a brief, explicit statement of the observational design and its limits (no comparator, self-pay, single-sex, six-month horizon) rather than leaving these solely to the Discussion.

Overall recommendation: Minor revision. The core dataset and findings are valuable and the rebuttal has substantially addressed most prior concerns.

Reviewer #2

(Remarks to the Author)

Reviewer #3

(Remarks to the Author)
The authors have provided a detailed response to the reviewer comments, and the revised manuscript is more clear and has additional details and supplementary tables that clarify the detailed methods, and limitations. This is a well written paper including over 8000 women started on a GLP1-RA and followed up for 6 months using a digital app as well as access to health care professionals and other staff when required. This is an innovative method of using the digital app to support patients and identify those that required additional support and outcomes at 6 months were as would be expected on such medications.
As a real world study, it does demonstrate feasibility in terms of service delivery in the appropriate context, outside conventional healthcare settings. However, several limitations have been highlighted, and these significantly impact the quality of the paper and the replicability in terms of wider rollout.
1. It was a pay for service model, although was reportedly discounted for the user (exact cost/discount not revealed but mention of it being cheaper than buying the drug itself as a private prescription). This does have an inherent selection bias and limitations in terms of wider rollout.
2. Follow up duration only 6 months, which is quite short in terms of long term weight management outcomes.
3. No active comparison group and observational nature of the study.
4. No blood pressure measurements or confirmation of other medications.

Open Access This Peer Review File is licensed under a Creative Commons Attribution 4.0 International License, which permits use, sharing, adaptation, distribution and reproduction in any medium or format, as long as you give appropriate credit to the original author(s) and the source, provide a link to the Creative Commons license, and indicate if changes were made.
In cases where reviewers are anonymous, credit should be given to 'Anonymous Referee' and the source.
The images or other third party material in this Peer Review File are included in the article's Creative Commons license, unless indicated otherwise in a credit line to the material. If material is not included in the article's Creative Commons license and your intended use is not permitted by statutory regulation or exceeds the permitted use, you will need to obtain permission directly from the copyright holder.
To view a copy of this license, visit https://creativecommons.org/licenses/by/4.0/

DETAILED RESPONSE DOCUMENT

EDITORIAL COMMENTS

Editor Comment 1:

We would recommend that, in addition to the reviewer points, the authors stress the novelty of the digital approach employed here. Some reviewers are concerned that other digital interventions for GLP-1 prescribing have been done previously.

Authors' Response:

We thank the Editor for this important direction and agree that the manuscript would benefit from clearer articulation of the novelty of our specific digital approach. We have revised the Introduction and Discussion to highlight the distinctive features of our programme relative to other direct-to-consumer and telehealth GLP-1RA platforms.

While digital prescribing of GLP-1 receptor agonists has been described in other publications, the majority of existing platforms operate as transactional prescribing services with limited additional features. Our programme has several integrated and protocol-driven components that, taken together, constitute a structured clinical-digital hybrid model that more closely replicates the safety and oversight features of specialist in-person care than transactional telehealth.

We have revised the manuscript to make this distinction explicit and have extended the platform description in the Supplementary Methods. We have also included two further references of relevant prior digital GLP-1RA datasets.

Editor Comment 2:

Please provide as much information as you can regarding the digital approach — any technical specifications of the app, time spent on remote calls, precisely how side-effects were handled if identified digitally, any technical/educational barriers identified in participants, how all information was validated for participants, anything relating to patient privacy and safety etc.

Authors' Response:

We have substantially expanded the programme description in both the Methods and Supplementary Methods. The revised text provides a detailed account of the following:

Platform architecture and access:

The programme was delivered entirely through a secure, access-controlled smartphone application. Participants could access live Proctor check-in sessions on demand through the application without requiring appointment scheduling. All data submitted through the platform were stored within a secure, audit-logged digital environment compliant with UK data protection legislation. Participant NHS GP details were collected during onboarding, and a summary of clinical information was shared with the participant's GP to support integrated care and safety oversight.

Side-effect identification and management:

Side effects could be reported through the in-app reporting function at any time or via a supplementary online form between scheduled contacts. In-app reports triggered immediate automated self-help guidance within the application; online form submissions prompted self-help advice by email. Reports classified as clinically significant or severe were escalated to the clinical team with a target review within one working day. Clinical review could result in tailored advice, dose modification, temporary treatment interruption, additional follow-up, or escalation to urgent care pathways where clinically indicated. All participants received a structured side-effect mitigation package at treatment initiation containing over-the-counter medications and dietary guidance targeting common GLP-1RA-associated gastrointestinal symptoms.

Data validation:

Participant identity was verified using photographic identification reviewed by a trained Proctor during a supervised live video onboarding session. Baseline weight was confirmed during the same supervised session. Subsequent weight measurements were performed using the same participant-owned digital scales used at onboarding, with participants instructed to measure under standardised conditions (light clothing, no footwear, consistent time of day). Medication adherence was verified through digital medication scanning and dose confirmation within the platform at weekly Proctor check-ins. Biochemical data were obtained from home capillary blood samples processed by Randox Clinical Laboratory Services, a UKAS-accredited laboratory, providing objective, externally validated metabolic measurements.

Educational access and engagement:

An evolving library of educational content was accessible to all participants throughout the programme, covering injection technique, nutrition, hydration, side-effect expectations and management, physical activity, and programme navigation. Content was delivered through articles, videos and live webinars, and was updated throughout participants' time on the programme to reflect emerging evidence and participant feedback.

Editor Comment 3:

Nature journals request that authors follow the SAGER guidelines and use the terms sex (biological attribute) and gender (shaped by social and cultural circumstances) carefully. If the research findings apply to only one sex or gender, that must be indicated in the title and/or abstract. The Reporting Summary should include whether sex and/or gender was considered in the study design and whether sex and/or gender of participants was determined based on self-report or assigned.

Authors' Response:

We have carefully reviewed the SAGER guidelines and revised the manuscript accordingly. The programme was designed exclusively for adult women (female sex at birth), and sex and gender were determined through structured self-report during onboarding, confirmed via supervised identity verification. As the study enrolled only one sex, no sex-disaggregated comparative analyses were appropriate or conducted.

Throughout the revised manuscript, we have used the term "female sex" when referring to the biological inclusion criterion, and "women" when referring to the cohort as a whole, consistent with the SAGER guidance. The title and abstract have been reviewed to ensure accurate and consistent use of terminology.

REVIEWER #1

Reviewer Comment 1-1:

The scale of the cohort and the exclusive focus on women are notable and address a genuine gap in the GLP-1 literature. However, the study is not methodologically novel. Similar real-world observational cohorts evaluating GLP-1RAs (including digital or routine-care pathways) have already been published. The principal contribution is therefore descriptive and implementation-focused, rather than hypothesis-testing or mechanistic. Significance is moderate, although would benefit from reframing as real-world implementation evidence rather than effectiveness evidence.

Authors' Response:

We are pleased that the reviewer recognised the "gap in the literature" which we have addressed with our study exclusively focused on women.

Our study is not intended as a mechanistic or "proof of effectiveness" investigation. The originality of our approach involved a bespoke digital platform to deliver clinical rigour without clinical visits. The challenge for GLP-1RA has been to extend evaluation and delivery to the large number of people in the population who might benefit outside the traditional healthcare systems (real-world implementation evidence). This study builds on reports of existing platforms which operate as transactional prescribing services with limited structured monitoring or safety oversight. These have often been small, retrospective, and often focused on diabetes. Our programme is distinguished by several integrated and protocol-driven components which have not been previously described in combination. These include:

- Mandatory monthly structured questionnaire completion as a condition of medication refill approval, ensuring continuous safety and tolerability monitoring;
- Weekly live video check-ins with trained Proctors providing real-time adherence verification, supervised weight confirmation using the participant's own validated scales, and medication scanning to confirm dose administration;
- A proactive, structured side-effect mitigation package dispensed at treatment initiation, accompanied by in-app self-help guidance and an escalation pathway to the clinical team within one working day;
- Monthly clinician-led review (by UK-registered GPs, Clinical Pharmacists and Advanced Nurse Practitioners) informing individualised, response-guided dose titration rather than automatic escalation;
- Mandatory home capillary blood testing at baseline and six months, processed by a UKAS-accredited laboratory (Randox), providing objective biochemical safety monitoring without clinic attendance;
- An evolving educational content library (articles, videos, live webinars) covering injection technique, side-effect self-management, nutrition and lifestyle, accessible at the point of need throughout the programme.

Taken together, these components constitute a structured clinical-digital hybrid model that more closely replicates the safety and oversight features of specialist in-person care than typical transactional telehealth. We have revised the manuscript to make this distinction explicit in the Introduction and Methods and have added a dedicated platform description to the Supplementary Methods. We also now reference two of the most relevant prior digital GLP-1RA datasets.

Reviewer Comment 1-2:

The observational cohort design is appropriate for service evaluation but cannot support causal claims about treatment effectiveness or equivalence to randomized trials. The absence of a comparator group (usual care, non-digital care, or lifestyle-only) substantially limits interpretability.

Authors' Response:

We agree with the reviewers point and have revised the manuscript to remove language that implies causal attribution or direct equivalence to randomised trial conditions. Phrases such as "trial-comparable outcomes" have been replaced with more precise language acknowledging that the observed weight reduction is consistent with the early-phase trajectories reported in tirzepatide and semaglutide RCTs, without implying formal equivalence.

The study was designed as a real-world service evaluation, and the primary question of interest was whether a structured digital delivery model could achieve clinically meaningful outcomes at scale in a community population, not whether it was superior or equivalent to any comparator. This distinction is now clearly stated in the revised Methods and Discussion.

We note that the broader evidence base for GLP-1RA pharmacotherapy includes robust randomised evidence for weight reduction. The contribution of the present study is to characterise real-world implementation outcomes in a population and setting which has not been represented in previous trials, not to re-establish drug efficacy.

Reviewer Comment 1-3:

The self-pay, self-enrolled model with subsidization during the study period introduces strong selection bias that the authors acknowledged. The fact that nearly half of the screened population was excluded might warrant comparison of baseline characteristics (which understandably may not be possible).

Authors' Response:

Individuals were only considered for inclusion in the analytic cohort if they completed baseline biochemical testing, as this was essential to evaluate treatment response. Of 16,121 individuals meeting initial screening criteria, 4,025 were not included because baseline blood testing was not completed. A further 308 were excluded following clinical review because of abnormal blood results or medical contraindications, and 3,661 were prescribed GLP-1RA therapy after the administrative inclusion cut-off date for this study.

Because detailed clinical and biochemical data were only available for individuals who completed baseline testing and clinical review, comparisons with non-included registrants were necessarily restricted to variables collected at initial online registration. We have added Supplementary Table 2 comparing the analytic cohort with the wider group of online registrants with valid baseline data. Baseline sociodemographic and anthropometric characteristics were broadly similar, with no major differences in age, baseline BMI, or socioeconomic status based on postcode-derived Index of Multiple Deprivation (IMD) quintiles.

The observed breadth of socioeconomic representation in the analytic cohort (37.6% of participants residing within the two most deprived IMD quintiles) and consistent outcomes across all deprivation strata provides some reassurance that participation was not restricted to the most advantaged groups.

Reviewer Comment 1-4:

Weight was measured using participant-owned scales and height was self-reported. While pragmatic, these data sources introduce measurement error that is not formally quantified or analysed. Approximately 20% of participants lacked paired biochemical data and ~10% lacked 6-month weight data.

Authors' Comment:

We agree that the sources of measurement error deserve more thorough discussion and sensitivity analysis:

Weight measurement:

Participant-owned digital scales were used throughout the programme. A design feature distinguishing this programme from simpler self-reporting models is that all participants used the same scales for every weight measurement, from the supervised baseline onboarding weigh-in through to all subsequent weekly Proctor check-ins. The baseline weight was confirmed by a trained Proctor via live supervised video, with photographic evidence where required. Subsequent measurements were verified at weekly Proctor sessions using video-supported weigh-ins. This longitudinal consistency reduces the inter-scale variability that would affect a programme relying on ad hoc self-reporting (described in the Supplementary Methods).

We additionally applied prespecified hierarchical data-cleaning procedures (described in the Supplementary Methods) to identify and exclude biologically implausible weight entries, including lb-to-kg transcription errors and isolated extreme deviations. Participants in the extreme tails of weight change (>=99th percentile) underwent manual review with comparison against proctored measurements.

Height was self-reported and used only to calculate baseline BMI. We acknowledge this as a limitation and note that self-reported height typically introduces a modest upward bias in reported stature, with a corresponding small underestimate of true BMI. This does not affect the primary weight-change outcome, which is based on longitudinal within-individual measurements using the same scales throughout follow-up.

Biochemical data:

Home capillary blood sampling using validated kits (Randox) processed by a UKAS-accredited laboratory represents a pragmatic but scientifically rigorous approach to biochemical monitoring at scale. Randox Clinical Laboratory Services operates to ISO 15189 accreditation standards with internal quality controls applied to each analytical run. The 20% incomplete paired biochemical rate primarily reflects logistical constraints inherent to home sample collection and postal return, rather than systematic clinical differences. Incompleteness occurred for reasons including sample haemolysis, insufficient sample volume, and postal delay, rather than participant health status.

Missing data:

We have added Supplementary Tables 3-4 comparing baseline characteristics between participants with and without complete paired weight and biochemical data respectively. Overall, participants with complete and incomplete paired outcome data were broadly comparable with respect to age, baseline weight and BMI, baseline biochemical measures, ethnicity, and socioeconomic status based on postcode-derived Index of Multiple Deprivation quintiles, providing limited evidence that missingness was strongly differential with respect to measured covariates. We also performed inverse probability weighted sensitivity analyses to assess the potential influence of missing outcome data. These are described in response to Comment 1-5 and presented in Supplementary Table 5.

Reviewer Comment 1-5:

All analyses are complete-case only, with no multiple imputation or inverse probability weighting.

Authors' Response:

We agree that additional sensitivity analyses were warranted to assess the potential influence of missing outcome data. The primary analyses remain complete-case analyses for each endpoint, including participants with paired baseline and follow-up measurements. However, we have now added inverse probability weighted sensitivity analyses for the primary and secondary paired change estimates.

For each outcome, the probability of having complete paired baseline and follow-up measurements was estimated using logistic regression. Models included age group, baseline BMI category, ethnicity, Index of Multiple Deprivation quintile, and baseline HbA1c as explanatory variables, with missing covariate values handled using missing-indicator categories. Stabilised inverse probability weights were calculated as the marginal probability of having complete paired outcome data divided by the predicted probability of having complete paired outcome data. Weight distributions were manually assessed for extreme values, and truncation was not required. Further details are provided in the Supplementary Methods.

These sensitivity analyses produced estimates materially similar to the primary complete-case analyses and are now presented in Supplementary Table 5. We have also added Supplementary Tables 3 and 4 comparing baseline characteristics between participants with and without complete paired weight and biochemical outcome data, respectively. The primary complete-case analyses and inverse probability weighted sensitivity analyses are now reported together in the revised manuscript. The conclusions remain based on the primary complete-case analyses, with the sensitivity analyses used to contextualise the potential impact of missing outcome data. We believe these additions provide greater transparency regarding how missing data may have influenced the main findings, while recognising that bias from unmeasured determinants of missingness cannot be excluded.

Reviewer Comment 1-6:

Subgroup analyses are exploratory but not corrected for multiplicity, increasing the risk of false-positive interpretation.

Authors' Response:

We agree that the subgroup analyses are exploratory and that multiplicity should be considered when interpreting formal subgroup comparisons. We have now applied the Benjamini-Hochberg false discovery rate (FDR) procedure to all secondary and exploratory analyses where related formal P values were derived. The relevant statistical methods, figure legends and results tables have been updated accordingly.

Following FDR correction, the overall interpretation of the subgroup analyses was not materially altered. The majority of statistically significant subgroup comparisons remained significant after adjustment. The Discussion has also been drafted to avoid overinterpreting specific subgroup findings and instead frames these analyses as contextual information on the range and consistency of treatment response.

Reviewer Comment 1-7:

The central descriptive finding—substantial short-term weight loss among retained participants—is internally consistent. However, conclusions frequently overreach, implying effectiveness "comparable to trials" and equity across socioeconomic strata without sufficient methodological support. Claims regarding safety, generalisability, and health equity are not robust given short follow-up, self-reporting, and selective inclusion.

Authors' Response:

We revised the Discussion and conclusions to remove or moderate language that overstates the strength of evidence. Specifically:

- "Trial-comparable outcomes" has been replaced with "weight reduction consistent with early-phase trajectories reported in tirzepatide and semaglutide randomised trials" without implying formal equivalence.
- Claims regarding health equity have been qualified: we now state that weight loss did not differ significantly across IMD quintiles in this cohort (P=0.364), which is a descriptive finding consistent with equitable access within the programme, but does not constitute evidence that digital pharmacotherapy does not exacerbate broader societal health inequalities in a general population context.
- Safety claims have been qualified to reflect the limited six-month follow-up period and the self-reported nature of adverse event data. We now state that no serious treatment-related adverse events were identified within the observation period, and that longer-term safety surveillance will be required.
- Generalisability statements have been tempered, with explicit acknowledgement of the predominantly White, self-selected, self-pay cohort. We have noted that the programme's design may not be directly transferable to NHS-funded or lower-income settings without further evaluation.

Reviewer Suggestions 1-8:

Reframe the manuscript explicitly as descriptive implementation research, avoiding causal or equivalence language

Authors' Response:

Thank you we have addressed this in the manuscript.

Reviewer Suggestions 1-9:

Provide a systematic comparison of included vs excluded participants, particularly those excluded due to incomplete labs.

Authors' Response:

Thank you we have addressed this in the manuscript with the addition of Supplementary Tables 3-4 described in Reviewer Comment 1-4.

Reviewer Suggestions 1-10:

Add a clear limitations box in the main text highlighting selection bias, measurement error, and commercial context.

Thank you. We appreciate the suggestion. We have addressed the key issues raised by the reviewer in an expanded standard limitations section. We would however be prepared to add a limitation box if the editors felt this would add value.

Reviewer Comment 1-11:

Key trials and recent real-world studies are cited appropriately. However, prior digital-health GLP-1 observational studies and direct-to-consumer critiques should be more explicitly discussed in the Introduction to contextualise novelty and limitations.

Authors' Response:

We have revised the Discussion to include a more explicit discussion of the existing landscape of digital and direct-to-consumer GLP-1RA prescribing. We contextualise our programme against these comparators, describing where our programme offers additional clinical governance, monitoring intensity, and structured support relative to simpler digital prescribing models. The limitations of the direct-to-consumer model, including selection bias, variable clinical oversight and concerns about inappropriate prescribing, are acknowledged.

REVIEWER #2

Reviewer Comment 2-1:

The authors should ensure comparison with relevant prior literature evaluating GLP-1RA use in digital or remote-care settings.

Authors' Response:

We have revised the Discussion to include comparison with the most relevant prior digital and remote-care GLP-1RA datasets. The three distinctive features of our programme relative to these comparators are: (1) mandatory monthly questionnaire completion as a condition of medication refill; (2) weekly live video Proctor check-ins with verified adherence and weight confirmation; and (3) mandatory home biochemical testing at baseline and six months. These components collectively represent a higher standard of real-world clinical monitoring than has been previously described for a digital GLP-1RA programme of comparable scale.

The primary comparators identified are:

- Johnson et al. (JMIR, March 2025): A retrospective cohort service evaluation from the Voy digital weight-loss platform (UK), reporting 5-month outcomes in adults using semaglutide or tirzepatide with app-based behavioural support. In contrast to our programme, the Voy model did not include mandatory biochemical monitoring, weekly Proctor-verified adherence, or a protocolised side-effect mitigation package.

- Ladebo et al. (Diabetes Care, 2024): A Danish cohort study characterising real-world semaglutide dose titration patterns outside of trial settings.

Reviewer Comment 2-2:

The absence of explicit exclusion criteria should be addressed. Participant characteristics indicate a predominantly healthy cohort with limited comorbidity, which restricts generalisability to broader populations who more commonly require GLP-1RA therapy due to obesity-related complications.

Authors' Response:

We have added a comprehensive statement of inclusion and exclusion criteria to the Methods section and Supplementary Methods of the revised manuscript. The full list of exclusion criteria includes: GLP-1RA use within the preceding 12 months; personal or family history of medullary thyroid carcinoma or multiple endocrine neoplasia type 2; history of pancreatitis or pancreatobiliary malignancy; active gallbladder disease; severe liver disease or ALT/AST >95 U/L; severe hypertriglyceridemia (>10 mmol/L); severe renal impairment; significant thyroid dysfunction (TSH <0.1 or >30 mIU/L); uncontrolled cardiovascular disease; type 1 diabetes; uncontrolled type 2 diabetes (HbA1c >100 mmol/mol); active malignancy; inflammatory bowel disease; pregnancy or breastfeeding; medications with a narrow therapeutic index; and active substance misuse or severe psychiatric instability. These are now presented in full in the Supplementary Methods.

We acknowledge the reviewer's observation that this cohort is comparatively healthy relative to populations seen in specialist obesity clinics. This reflects both the programme's community-based direct-access model and its safety-oriented exclusion criteria, as well as the way that these drugs are likely to be used in the future. The mean baseline HbA1c of 36.2 mmol/mol, type 2 diabetes prevalence of 2.5% and modest prevalence of established cardiovascular disease are consistent with a community, self-selected population rather than a tertiary referral population. We have acknowledged in the Discussion that generalisability to populations with more advanced comorbidity cannot be assumed from the present data, and that prospective evaluation in these groups will be required.

Reviewer Comment 2-3:

Clarify whether participants received regular clinical follow-up, physician or dietitian consultation, or wraparound care.

Authors' Response:

We have substantially expanded the description of clinical oversight in the revised Supplementary Methods. All participants received structured monthly clinical review by UK-registered clinicians (General Practitioners, Clinical Pharmacists, and Advanced Nurse Practitioners) prior to each medication refill. This review incorporated weight trajectory data, side-effect reports, medication adherence information, refill questionnaire responses, and any new clinical concerns. Clinical oversight was provided by a pooled team of 25 clinicians. Dosing decisions, including escalation, maintenance, or modification, were made at each monthly review on an individualised basis. Ad hoc clinical review was also available for participants reporting significant side effects, with a target response time of one working day for escalated concerns.

In addition to clinical review, participants had access to a 10-week structured online lifestyle programme, developed in conjunction with dietitians and physiotherapists, which could be accessed in a self-directed

manner. Participants did not have direct synchronous access to dietitians or physiotherapists, but the educational content was professionally developed and covered nutrition, physical activity and behavioural strategies relevant to weight management. NHS GP details were collected mandatorily at enrolment, and clinical information was shared with the participant's own GP to support integrated care.

Reviewer Comment 2-4:

Discuss whether programme fees affected uptake or demographic skew, particularly the underrepresentation of underserved communities.

Authors' Response:

This issue goes to the heart of scalability and equity. The programme operated as a fixed-fee self-pay service covering pharmacotherapy, laboratory testing, the structured app-based monitoring pathway, and clinical oversight components. During the study period, programme fees were partially subsidised by the provider, making the total cost to participants substantially lower than the prevailing market rate for GLP-1RA therapy obtained through private prescription in the UK.

We acknowledge that a self-pay model introduces an inherent economic access barrier, and that willingness to pay for a structured health programme may itself select for individuals with higher health motivation, a form of motivation-related selection bias that may contribute to the high retention and favourable outcomes observed. This is now explicitly acknowledged in the limitations section.

Despite this concern, the socioeconomic distribution of the analytic cohort was notably broad: 37.6% of participants resided within the two most deprived IMD quintiles, suggesting that the subsidised pricing model and digital-first access (obviating the need to travel to clinic, take time off work, or navigate national referral pathways) may have partially mitigated economic barriers. Importantly, weight loss, metabolic outcomes, side-effect burden and retention did not differ significantly across IMD quintiles, suggesting that once enrolled, participants from more deprived backgrounds achieved outcomes equivalent to those from less deprived backgrounds. This is a meaningful and novel finding and we have highlighted more prominently in the revised Discussion. During the study, we asked participants additional questions about their lifestyle and in particular changes to their spending patterns including food, alcohol and smoking. These findings will be reported separately in view of the potential cost savings to the individual which could result from these therapies.

We acknowledge that the programme's current pricing structure would not be sustainable as a universal access model without public subsidy, and that equitable access at scale would require integration with national commissioning pathways or targeted subsidy for lower-income groups. The landscape for these incretin-based weight loss therapies is changing rapidly, including oral administration and cost reductions due to patent loss. This will affect the economics for the individual and the healthcare systems and these considerations are now discussed.

Reviewer Comment 2-5:

Describe any mechanisms used to validate self-reported outcomes or, if none, more fully acknowledge this limitation.

Authors' Response:

Validation mechanisms were an integral part of the programme design and are now described in the revised Supplementary Methods. The key validation components were:

- Weight: Baseline weight confirmed by trained Proctor via supervised live video. All subsequent weights measured using the same participant-owned digital scales used at baseline onboarding, with weekly Proctor video check-ins providing ongoing verified weight monitoring throughout the programme.

- Identity and enrolment information: Identity verified using photographic identification during supervised video onboarding. NHS GP details were collected mandatorily, providing an additional layer of accountability.

- Biochemical data: Objective, externally validated measurements from home capillary blood samples processed by a UKAS-accredited laboratory (Randox Clinical Laboratory Services, ISO 15189 accredited). All samples were returned via pre-paid priority biological specimen postal packaging compliant with UK transport regulations.

- Medical history and medications: Self-reported and not externally verified against NHS records, reflecting the limitations of a private direct-access service model. This is acknowledged as a limitation. However, participant-reported medical history was shared with the participant's own GP, who could advise the programme of any inaccuracies, providing a partial verification mechanism.

We have added wording to the limitations section that medical history and medication data were self-reported and not systematically verified against external clinical records.

Reviewer Comment 2-6:

Explain the absence of a 12-month follow-up period. Weight trajectories with GLP-1RA therapies often peak or plateau around 12 months and omission of this time point limits understanding of maximal effect and durability.

Authors' Response:

The six-month observation horizon reflects the duration of available follow-up at the time of this analysis. Twelve-month data is therefore not yet available at the time of submission. We agree that six months captures the early-phase trajectory of GLP-1RA-mediated weight loss and that maximal pharmacodynamic effect and durability of response require longer follow-up.

The programme is ongoing and all participants are being followed longitudinally. Analysis of 12-month data, including weight trajectory durability, biochemical outcomes, dose-response relationships and predictors of long-term retention and weight maintenance, is planned and will form the basis of subsequent publications. We have added a statement to this effect in the Discussion, alongside acknowledgement that the six-month data, while important and novel, do not address the questions of maximal effect or long-term durability that will be critical for health economic modelling and commissioning decisions.

REVIEWER #3

Reviewer Comment 3-1:

Lack of data on blood pressure is noted, especially when discussing cardiometabolic risk. Follow up at 6 months is not long enough to determine long term outcomes but does suggest this is potentially a feasible long-term model of care.

Authors' Response:

Blood pressure was not collected as part of the programme's routine digital data capture during the study period. We have acknowledged this as a limitation in the revised Discussion, noting that blood pressure is a key cardiometabolic risk marker and that its absence limits our ability to characterise the full cardiovascular risk profile of the cohort at baseline or to assess the programme's impact on hypertension outcomes.

The development of home blood pressure monitoring as a standard component of the digital care pathway is an identified priority for future iterations of the programme, and we note this as a direction for future work. We also note that waist circumference, a marker of visceral adiposity that provides additional cardiovascular risk information beyond BMI, was similarly not collected and has been added to the limitations discussion.

Regarding the six-month follow-up horizon, we agree with the reviewer's characterisation: the present data support the feasibility of the model rather than establishing long-term outcomes. We have moderated the language in the Discussion accordingly, while noting that the magnitude of weight loss and metabolic improvement at six months is clinically meaningful and provides a robust basis for the planned 12-month analysis.

Reviewer Comment 3-2:

Would the authors be able to expand on what amount was paid, and how this compares to simply buying the medication with a prescription from their primary care physician? There is an inherent selection bias in such a program that includes people who can afford to self pay. Cost of the program itself in addition to the cost to the participant is also a major factor in determining whether or not such a program is scalable and sustainable.

Authors' Response:

We thank the reviewer for raising this important practical consideration. During the study period, programme fees were partially subsidised by the provider, with the aim of making access available across a broad range of socioeconomic backgrounds. The total fee covered pharmacotherapy (tirzepatide or semaglutide), baseline and six-month home capillary blood test kits (with accredited laboratory processing), the structured app-based monitoring pathway including weekly Proctor access and monthly clinical review, and the side-effect support package. The subsidised fee was set at a level substantially below the prevailing market rate for private GLP-1RA prescriptions in the UK.

In the UK, individuals meeting NICE criteria (TA1026) for tirzepatide are eligible for NHS-funded treatment through specialist tier 3 weight management services. However, access to these services is severely constrained by capacity limitations, with long waiting lists in most NHS trusts. The programme therefore served a population unable or unwilling to access NHS pathways, at a price point that was lower than equivalent private prescription costs. The initial blood test kit cost (£49) was refunded if the participant was found to be ineligible, further reducing the financial risk to potential participants.

We acknowledge, as the reviewer correctly identifies, that a self-pay model is not inherently scalable or equitable, and that the subsidised pricing during this period may not be a sustainable commercial structure.

We have added discussion of these issues. The broad IMD distribution of the cohort suggests that the pricing and digital-access model was not exclusionary, even at its current level.

Reviewer Comment 3-3:

While pregnancy was excluded at baseline, was it also discussed in the interim visits alongside contraception? It would be more important in a woman-only study, given that these medications are not safe in pregnancy. Were there any pregnancies reported in the study duration?

Authors' Response:

Pregnancy status and contraception were addressed both at baseline enrolment and throughout the programme. At onboarding, pregnancy and breastfeeding were explicit exclusion criteria. Ongoing pregnancy status was assessed at each monthly review cycle through the structured refill questionnaire. Participants were advised to use effective contraception if of childbearing potential and to contact the clinical team immediately if pregnancy was suspected or confirmed during the programme. Programme guidelines specified immediate treatment cessation and clinical escalation in the event of pregnancy. Regarding pregnancies during the study period a small number of participants (11) reported pregnancy or intention to conceive during the six-month observation period, all of whom discontinued treatment in accordance with programme protocols. We have included this in the Results and Supplementary Methods.

Reviewer Comment 3-4:

There is a lack of detail of medications being taken, particularly in type 2 diabetes and dyslipidaemia. Were there any changes in medications during the study, and were there any additional (or fewer) medications taken that may have influenced HbA1c and lipid outcomes?

Authors' Response:

Concomitant medication data were captured at baseline and requested at each monthly refill questionnaire through participant self-report. We acknowledge that self-reported medication data in a direct-access private programme are not systematically verified against prescription records and have added this limitation in the revised manuscript. However, reported use of glucose and lipid-lowering therapies was uncommon, and newly started medications in these classes were infrequently reported during follow-up. This makes it unlikely that concomitant medication changes fully accounted for the observed HbA1c and lipid outcomes, although residual misclassification or unreported medication changes cannot be excluded. This has now been added to the Results and Discussion of the revised manuscript, and the underlying data has been submitted as Supplementary Data 1. Metabolic outcome analyses should therefore be interpreted as reflecting the aggregate effect of the programme (weight loss plus any concomitant medication changes) rather than isolating the pharmacodynamic effect of GLP-1RA therapy per se.

Reviewer Comment 3-5:

Was there a specific rationale behind use of >2 kg/month as a reason not to uptitrate medication, as this was not used in the clinical trials, and the baseline weight/BMI of patients in this study was 38.2?

Authors' Response:

The threshold of >=2 kg/month weight loss as a criterion to defer dose escalation was derived from NHS and evidence-based obesity management guidance promoting gradual and sustainable weight loss, which is consistently described within UK clinical guidelines as approximately 0.5–1 kg per week (equivalent to approximately 2–4 kg per month). The lower bound of this range (0.5 kg/week x 4 weeks = 2 kg/month) was adopted as the programme threshold to provide clinicians with a practical reference point for response-guided titration, consistent with established UK practice.

The rationale for this approach was to avoid unnecessary escalation in participants already achieving clinically meaningful weight loss, thereby minimising dose-dependent side-effect burden and the risk of unnecessary gastrointestinal adverse effects. Given that mean tirzepatide exposure at six months was 9.5 mg/week, substantially below the licensed maximum of 15 mg/week, and that 83.1% of participants achieved >=10% weight loss, this response-guided titration strategy appears to have been effective without requiring maximal dosing in most participants. Clinical trial dosing strategies are optimised for maximal efficacy, whereas the programme's approach was designed to balance effectiveness with tolerability and medication efficiency in a real-world setting. This distinction is now noted.

Reviewer Comment 3-6:

For HbA1c, in addition to mmol/mol, it would be helpful to also have % values.

Authors' Response:

We agree that presenting HbA1c in both IFCC mmol/mol and NGSP % units will improve interpretability for readers familiar with either reporting convention. In the original manuscript, HbA1c values were presented in mmol/mol in line with SI reporting. We have now revised the main Results text to report HbA1c values in both mmol/mol and NGSP % units for key findings. Figures continue to present HbA1c in mmol/mol for consistency.

Reviewer Comment 3-7:

In Table 5, perhaps it is better to use Tirzepatide rather than Mounjaro to be consistent with the rest of the manuscript.

Authors' Response:

We thank the reviewer for this observation. All instances of "Mounjaro" in Table 5 and throughout the manuscript have been replaced with "tirzepatide" in keeping with standard scientific reporting practice.

Digital-first delivery of GLP-1 receptor agonist therapy in a large real-world cohort of women

DETAILED RESPONSE DOCUMENT – 22 July 2026

REVIEWER #1

We are pleased that Reviewer 1 felt that we have now addressed the major issues that had been raised from the review process. We provide below a point-by-point response to the outstanding comments.

Reviewer Comment 1: Summary of Key Results

This is a real-world observational service evaluation of 8,127 women initiating GLP-1RA therapy (99.8% tirzepatide) through a UK digital programme, with six-month follow-up. Key findings: mean weight loss of 16.1 kg (15.5%), with 83.1% achieving ≥10% loss; substantial HbA1c and lipid improvements; 91.4% retention; no serious treatment-related adverse events.

Authors' Response:

No response required.

Reviewer Comment 2: Originality and significance

The exclusive focus on women is a genuine and useful contribution. The authors' response letter reframes the contribution as "implementation evidence" rather than effectiveness evidence, which I agree is the more defensible framing and should be reflected more consistently in the abstract. The novelty rests on the bundle of monitoring components (mandatory labs, weekly video check-ins, structured titration) in my opinion.

Authors' Response:

We thank the Reviewer and have revised the abstract to reflect all the points made.

Reviewer Comment 3: Data & methodology: validity of approach, IPW weighting for missingness

The authors describe stabilised IPW models for probability of complete paired outcome data, using age group, baseline BMI category, ethnicity, IMD quintile, and baseline HbA1c as predictors. This approach can only correct for missingness that is missing at random (MAR) conditional on these observed covariates. It cannot correct for missing-not-at-random (MNAR) mechanisms, which are highly plausible here. I think it's a bit much to try and do more sensitivity analyses for MNAR because I don't think the overall message will change, but something to consider.

The rebuttal states IPW-weighted estimates were "materially similar" to complete-case estimates. This is presented as reassurance, but if the missingness model has limited discriminative power (i.e., the included covariates weakly predict missingness), IPW and complete-case estimates would converge. The authors should report a measure of the missingness model's discriminatory ability (e.g., c-statistic) if they haven't. I wasn't able to find the supplementary methods.

Authors' Response:

We thank the Reviewer for this important point. We agree that inverse probability weighting can address missingness only under the missing at random (MAR) assumption (conditional on the observed covariates). However, our approach cannot account for missing not at random (MNAR) mechanisms.

In response, we have added the c-statistic for each model predicting outcome completeness and summaries of the corresponding stabilised weight distributions to Supplementary Table 5. The c-statistics ranged from 0.600 to 0.684, indicating modest discrimination, while the stabilised weights showed no evidence of extreme values or instability. We have also revised the Results to report these diagnostics and the discussion to clarify the conditional MAR assumption and the potential for residual bias under MNAR.

The IPW estimates remained materially similar to the complete-case estimates across the primary and secondary weight and biochemical outcomes. This consistency provides some reassurance regarding the robustness of the findings under the MAR assumption. However, the potential impact of MNAR mechanisms cannot be evaluated using the available data without imposing additional, unverifiable assumptions, and we now acknowledge this explicitly as a limitation.

Reviewer Comment 4: Appropriate use of statistics and treatment of uncertainties

It seems like hazard ratios for IMD quintiles 2 and 5 not differing significantly from quintile 1, while quintiles 3–4 do, is an unusual, non-monotonic pattern that is not discussed — this should be commented on as it slightly undercuts the no meaningful socioeconomic gradient narrative.

Authors' Comment:

We thank the Reviewer for highlighting this point. We agree that the association between IMD and treatment discontinuation was non-monotonic and did not demonstrate a clear socioeconomic gradient. We have revised the Results to describe this pattern explicitly, noting that discontinuation hazards were lower in IMD quintiles 3–4 than in the most deprived quintile, while no significant differences were observed for quintiles 2 and 5. We have also added a sentence to the discussion clarifying that the similar hazards observed in the most and least deprived groups do not support a simple linear socioeconomic gradient.

Reviewer Comment 5: Conclusions: robustness, validity, reliability

No additional comments.

Authors' Response:

No response required.

Reviewer Comment 6: Suggested Improvements

Report the IPW weight distribution and missingness-model discrimination statistic (as above).

Discuss the non-monotonic IMD gradient in discontinuation hazard.

Authors' Response:

We have addressed these points in the manuscript and provided specific responses in comments 3 and 4.

Reviewer Comment 7: References

No additional comments

Authors' Response:

No response required.

Reviewer Suggestions 8: Clarity and Context

The manuscript is clearly written and well-organised. The abstract could be strengthened by including a brief, explicit statement of the observational design and its limits (no comparator, self-pay, single-sex, six-month horizon) rather than leaving these solely to the Discussion.

Authors' Response:

Thank you for your review of our manuscript and we have revised the paper to reflect all the points made.

REVIEWER #3

Reviewer Comment 1:

The authors have provided a detailed response to the reviewer comments, and the revised manuscript is more clear and has additional details and supplementary tables that clarify the detailed methods, and limitations. This is a well written paper including over 8000 women started on a GLP1-RA and followed up for 6 months using a digital app as well as access to health care professionals and other staff when required. This is an innovative method of using the digital app to support patients and identify those that required additional support and outcomes at 6 months were as would be expected on such medications.

As a real-world study, it does demonstrate feasibility in terms of service delivery in the appropriate context, outside conventional healthcare settings. However, several limitations have been highlighted, and these significantly impact the quality of the paper and the replicability in terms of wider rollout.

1. It was a pay for service model, although was reportedly discounted for the user (exact cost/discount not revealed but mention of it being cheaper than buying the drug itself as a private prescription). This does have an inherent selection bias and limitations in terms of wider rollout.

2. Follow up duration only 6 months, which is quite short in terms of long-term weight management outcomes.

3. No active comparison group and observational nature of the study.

4. No blood pressure measurements or confirmation of other medications.

Authors' Response:

We have not responded to Reviewer 3's comments as directed by your Editorial Response.
